# Reciprocal Sign Epistasis between Frequently Experimentally Evolved Adaptive Mutations Causes a Rugged Fitness Landscape

**DOI:** 10.1371/journal.pgen.1002056

**Published:** 2011-04-28

**Authors:** Daniel J. Kvitek, Gavin Sherlock

**Affiliations:** Department of Genetics, Stanford University, Stanford, California, United States of America; University of Michigan, United States of America

## Abstract

The fitness landscape captures the relationship between genotype and evolutionary fitness and is a pervasive metaphor used to describe the possible evolutionary trajectories of adaptation. However, little is known about the actual shape of fitness landscapes, including whether valleys of low fitness create local fitness optima, acting as barriers to adaptive change. Here we provide evidence of a rugged molecular fitness landscape arising during an evolution experiment in an asexual population of *Saccharomyces cerevisiae*. We identify the mutations that arose during the evolution using whole-genome sequencing and use competitive fitness assays to describe the mutations individually responsible for adaptation. In addition, we find that a fitness valley between two adaptive mutations in the genes *MTH1* and *HXT6/HXT7* is caused by reciprocal sign epistasis, where the fitness cost of the double mutant prohibits the two mutations from being selected in the same genetic background. The constraint enforced by reciprocal sign epistasis causes the mutations to remain mutually exclusive during the experiment, even though adaptive mutations in these two genes occur several times in independent lineages during the experiment. Our results show that epistasis plays a key role during adaptation and that inter-genic interactions can act as barriers between adaptive solutions. These results also provide a new interpretation on the classic Dobzhansky-Muller model of reproductive isolation and display some surprising parallels with mutations in genes often associated with tumors.

## Author Summary

How organisms adapt to their environment is of central importance in biology, but the molecular underpinnings of adaptation are difficult to discover. Fitness landscapes illustrate possible steps adaptive evolution can take to increase the evolutionary fitness of individuals within a population, and the shape of the fitness landscape determines the accessibility of the fittest point on the landscape. On a rugged landscape, negative interactions between mutations cause fitness valleys separating fitness peaks, which can constrain adaptation and act as an adaptive barrier. Here, we comprehensively characterized the fitness of mutations that arose in clones during a yeast experimental evolution and found that mutations in two loci, *MTH1* and *HXT6/HXT7*, arose multiple times independently and are individually adaptive. However, when forced to co-occur, the double mutant has a lower fitness than either single mutant and even the wild-type strain. This negative interaction forces these two mutations to remain mutually exclusive during the experimental evolution and results in a rugged fitness landscape, where genetic constraint prevents lineages carrying the *MTH1* mutation from reaching the higher fitness peak of *HXT6/HXT7*. These results show that genetic interactions are central in shaping a very active portion of this fitness landscape.

## Introduction

Introduced by Wright, the fitness landscape describes the possible mutational trajectories by which lineages evolve in a stepwise manner from genotypes that lie in regions of low fitness to ones of higher fitness [1], [2]. When viewed as a whole, this metaphorical landscape represents a species' possible paths of adaptive evolution towards the optimal genotype in a particular environment. An outstanding question is whether the selective surface of fitness landscapes is smooth, containing a single global fitness optimum, or rugged, where selective constraints on differing mutational trajectories create multiple local fitness optima [3]–[7]. If the landscape is smooth, any path leading to the optimal genotype that continuously increases the population's fitness will be selectively favored, and the population will reach the global optimum on the landscape. However, if the landscape is rugged, adaptation will be constrained by the mutations available to increase the population's fitness [3]. This ruggedness can hamper the efficacy of natural selection compared to a smooth landscape by slowing the rate of adaptation due to pervasive genetic constraint [8].

Genetic constraint on fitness landscapes is due to fitness epistasis, where a mutation's adaptive value depends on the genetic background in which it arises [6]. Epistasis is a key component in such processes as reproductive isolation and speciation [9], [10], the evolution of sex and recombination [11], [12], as well as human diseases [13]. Theory shows that a form of epistasis called “sign epistasis” is necessary to constrain mutational trajectories on fitness landscapes [14]. Sign epistasis occurs when mutations are beneficial within the context of some genetic backgrounds, but detrimental within others. However, it is an extreme form of sign epistasis, recently dubbed “reciprocal sign epistasis”, that is necessary to create the local peaks and valleys on fitness landscapes [7], [14], [15]. Reciprocal sign epistasis occurs when the mutational path between two genotypes is selectively inaccessible due to intermediate, low-fitness genotypes. Such valleys are less likely to be crossed by natural selection alone, depending on the mutation rate and population size [16]. Therefore, genotypes that reside at local fitness optima are likely dead-ends for natural selection: even if a higher fitness peak exists elsewhere on the landscape, the neighboring fitness valley impedes adaptation to the global fitness optimum.

Experimental studies aimed at testing mutational constraint on fitness landscapes due to epistasis have focused on testing engineered, biased amino acid substitutions, such as mutating residues at enzymes' active site(s) [17], [18], engineering likely evolutionary intermediates between ancestral and adapted versions of a single protein [19]–[21], or quantifying interactions between mutations in different genes that display an observable phenotype [22], [23]. Such work suggests that genetic constraint due to sign epistasis is prevalent and that adaptation can take surprisingly few mutational paths to the optimal genotype on the landscape. Some genotype-phenotype mapping studies using molecular data have inferred a multi-peaked landscape using proxies for fitness, but the extent of the role played by local optima during adaptation was either unknown [23] or limited [17], [24].

Here we describe a rugged fitness landscape that arose during an experimental evolution. We identify the molecular nature of the mutations resulting from the evolution, and describe which mutations are individually adaptive. Adaptive mutations in two genes appear several times in different adaptive lineages, and we determine that they are selectively mutually exclusive due to reciprocal sign epistasis. The genetic constraint between these two mutations causes a rugged fitness landscape, as both mutations occur multiple times during the evolution and are highly adaptive individually, while highly maladaptive in concert. This work shows that inter-genic interactions can act as barriers between adaptive solutions and adds to the mounting experimental evidence that the constraint caused by epistasis is of central importance in evolutionary biology.

## Results

### Whole-genome sequencing of experimentally evolved clones reveals novel mutations

**Figure 1:**
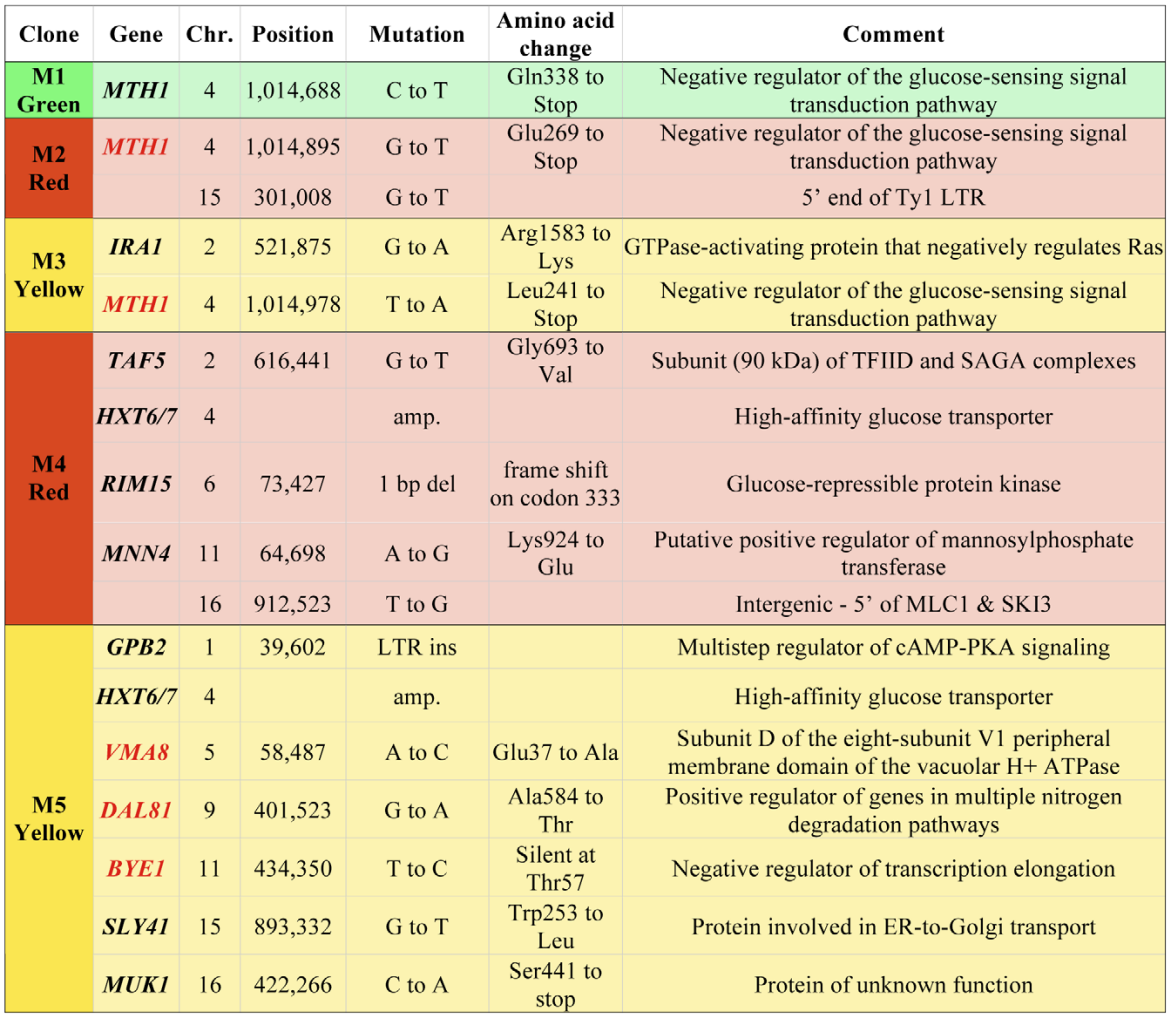
Mutations in adaptive clones M1–M5. Clones are colored according to their colored subpopulation of origin. New mutations found by whole genome sequencing are highlighted with gene names in red.

We have further characterized a previously described population of asexually-propagated haploid *S. cerevisiae* that were experimentally evolved under glucose limitation in continuous culture for 448 generations [25]. In that study, the chemostat was seeded with equal quantities of three otherwise isogenic haploid S288c strains that each expresses a different fluorescent protein constitutively (GFP, YFP or DsRed), and the proportions of the three colored lineages were tracked over time using flow cytometry. Expansions and contractions of the colored subpopulations were monitored, and a total of five adaptive clones (M1–M5), were isolated from the various colored subpopulations at generations 56, 91, 196, 266 and 385, respectively (see Materials and Methods for additional details on experimental design). That study identified a total of 12 independent mutations in these clones, using tiling microarrays [25]. However, through analysis of progeny of these clones, we discovered that some of them harbored additional, adaptive mutations of unknown identity, so we performed whole-genome sequencing on all five clones and their ancestor in order to identify these mutations. Sequence coverage of the nuclear genome ranged from 21× to 45× (Table S1). As expected, we discovered additional mutations: a total of five additional single nucleotide polymorphisms (SNPs) in M2, M3 and M5 (Figure 1). All SNPs, indels and copy number variants found previously using tiling microarrays [25] were detected with this genome sequencing approach, though our sequence data were not able to discover the LTR insertion in *GPB2* in M5 that we previously characterized, which is likely a limitation of the single-end sequencing.

We also estimated the copy number of the *HXT6/7* amplifications in M4 and M5 using real-time quantitative PCR (qPCR), targeting the nearly identical *HXT6* and *HXT7* coding regions as well as the *HXT7* promoter. These data were normalized to an ancestral strain without the amplification, which has one copy each of the *HXT6* and *HXT7* ORFs flanking the *HXT7* promoter (see Figure S1 for the region's structure). Given the model of mitotic recombination proposed by [26] to explain the *HXT6/7* amplification, the number of *HXT6/7* ORFs should be one greater than the number of *HXT7* promoter regions, regardless of the number of amplifications that have occurred. The qPCR results indicate that there are ten *HXT6/7* ORFs and nine *HXT7* promoters in M4, while M5 has 8–10 *HXT6/7* ORFs and 7–9 *HXT7* promoters (Figure S2). These data suggest there were a minimum of four mitotic recombination events for M4 and three for M5 to produce each array of *HXT6/7* genes. Comparing these results to the sequencing coverage of the *HXT6/7* region show that the coverage-based analysis underestimated the copy number for both clones (Figure S1), which may be due to the mapping algorithm used.

### Fitness characterization of individual mutations in evolved clones

**Figure 2:**
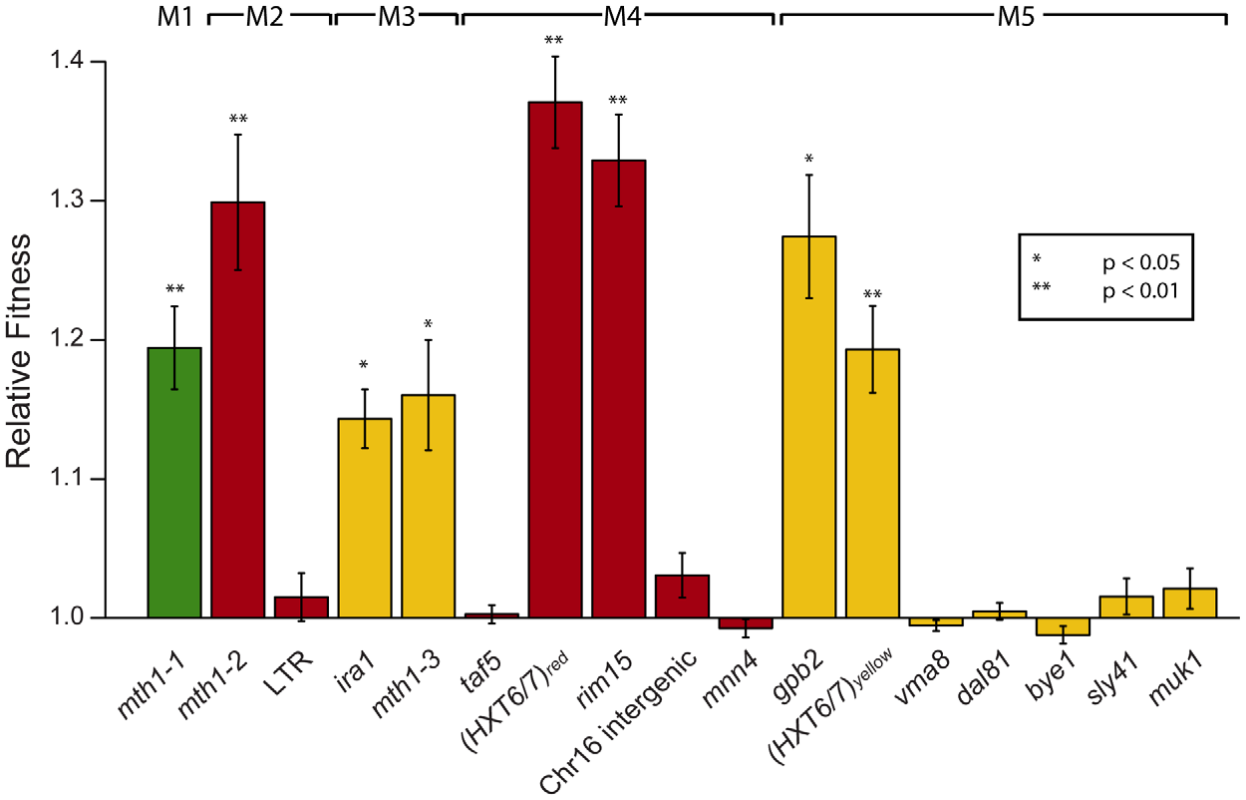
Relative fitness of individual mutations derived from competition experiments. Mutations are ordered as in Figure 1, with M1–M5 going left to right, and bars are colored according to the color subpopulation of origin of each clone. Error bars are +/− standard error of the mean. Significance was determined by a separate two-sample, two-tailed t-test for each mutation versus the wild-type control. (*) indicates p<0.05, (**) indicates p<0.01.

Each mutation could be the result of positive natural selection, or alternatively, could be a neutral or slightly deleterious mutation that hitchhiked along with one or more adaptive mutations. Thus, we segregated the mutations and determined each one's fitness effect in genetic isolation using competition experiments [27], where the only genetic difference between the mutant and wild-type competitor strain was the single mutation. Surprisingly, these data indicate that only 1–2 mutations per clone confer a significant fitness increase when considered singly, regardless of the total number of mutations in an adaptive clone (Figure 2). This increase in the number of seemingly non-adaptive mutations in later clones (M4 and M5) could be due to a lack of sensitivity in our single mutation fitness assay, an accumulation of neutral or deleterious hitchhiking mutations (Muller's ratchet [28]), or to non-additive fitness effects between mutations (positive, synergistic epistasis [6]). Given the 1.2×10^7^ bp size of the *S. cerevisiae* genome, a mutation rate of 5.12×10^−10^ bp^−1^ generation^−1^ [29], and the number of generations passed for these evolved clones, the expected number of neutral mutations is 1.6 for M4 and 2.4 for M5, while the probability of accumulating 4 or more neutral mutations in M4 is 0.084 and 5 or more in M5 is 0.092 (Poisson distribution). While these probabilities are low, they do not allow us to reject the null hypothesis that these mutations are neutral. Thus, our results are consistent with there being 1–2 adaptive mutations per clone, with the remaining mutations being neutral, though we cannot unequivocally rule out there being additional adaptive mutations in M4 and M5. Notably, we have also observed a nonsense mutation in *MUK1* in an independent evolution experiment performed under glucose limitation (Wenger and Sherlock, unpublished), suggesting that it may be adaptive either singly or in concert with another mutation.

**Figure 3:**
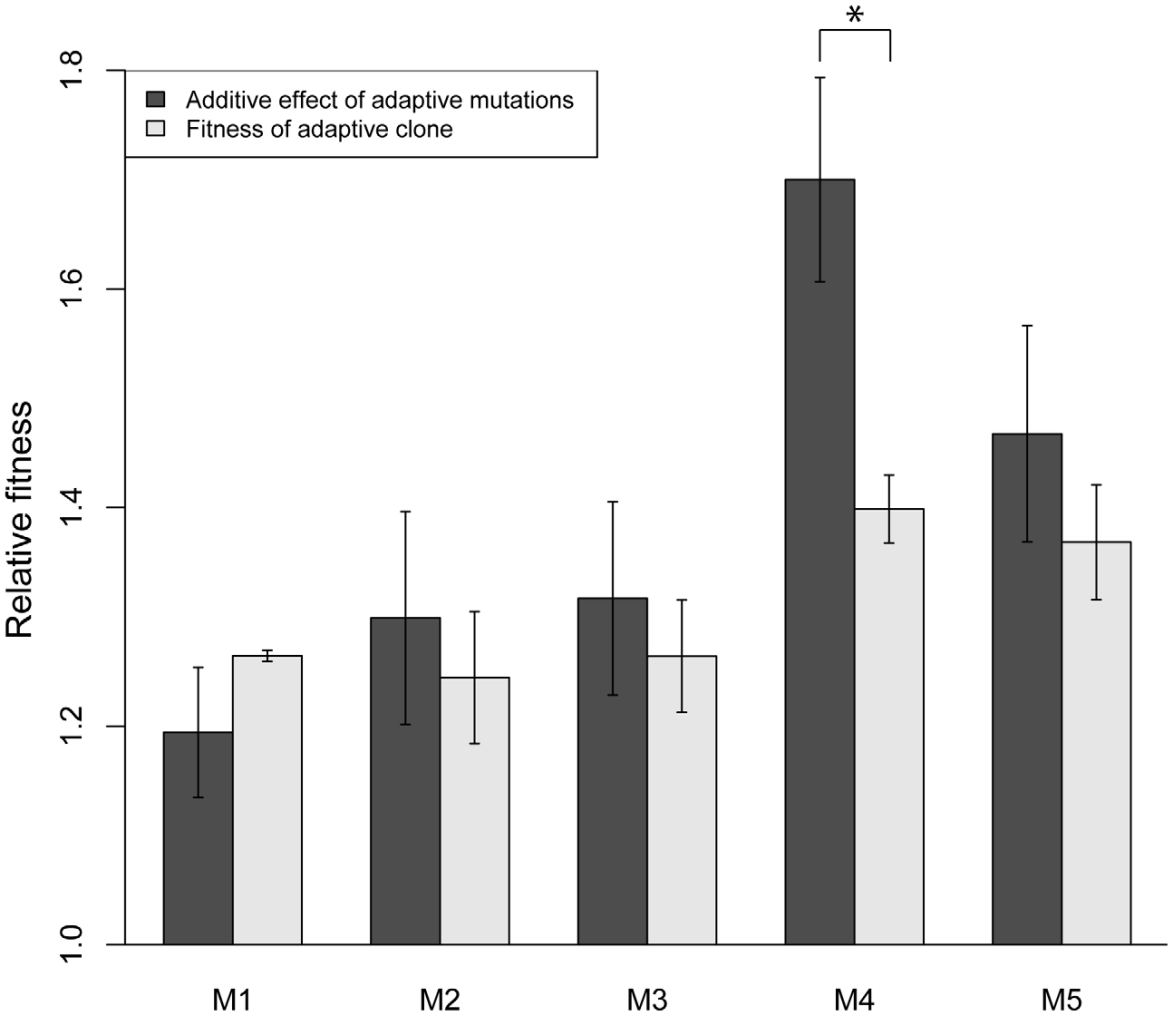
Adaptive mutations recapitulate fitness of adaptive clones M1–M3 and M5. For each adaptive clone, the fitness effects of each adaptive mutation from Figure 2 were added and compared to the relative fitness of the clone. The additive effects recapitulate the relative fitness of clones M1–M3 and M5, and there is evidence of negative epistasis between the two adaptive mutations in M4, since the additive fitness effect of the mutations is significantly larger than the relative fitness of the clone. Error bars are standard deviation. (*) indicates significance at α = 0.05. Relative fitness data of clones are from [25].

To test for evidence of additional adaptive mutations, we calculated the sum fitness effect of all the singly adaptive mutations from a particular clone to determine if that sum recapitulates the overall fitness of the clone. If additional adaptive mutations exist, the sum effect will be less than the fitness of the clone, assuming a no epistasis model. We found that the additive fitness effects of the individual adaptive mutations recapitulated the fitness of adaptive clones M1–M3 and M5 (Figure 3). In M4, the additive fitness effect of the two adaptive mutations is significantly larger than the fitness of the adaptive clone. This suggests that negative, antagonistic epistasis between two mutations in M4 causes a reduction in its overall fitness. This may be because the fitness effect of each individual mutation is so large that when combined using an additive model, the additive fitness effect is larger than the upper bound of fitness in the given environment.

The genes containing singly adaptive mutations are enriched [30] for the GO terms “hexose transport” and “negative regulation of Ras protein signal transduction” (p = 1.36e-4 and p = 5.8e-3, respectively; FDR<0.01%), suggesting that adaptation was due to increased glucose transport and signaling through the Ras/cAMP pathway, as previously hypothesized [25]. We compared the mutations found in our experiment with *S. cerevisiae* polymorphism data [31], and found that *MTH1* and *RIM15* both have naturally-occurring premature stop codons alleles in environmental isolates. This suggests that the mutations leading to premature stop codons we see in these genes during experimental adaptation to limiting glucose are also ecologically-relevant mutations that may provide a fitness advantage in nature. Furthermore, we have also observed variation in the copy number of the *HXT6/HXT7* locus in a survey of ∼70 yeast strains (B. Dunn and G. Sherlock, unpublished), again suggesting ecological relevance of these mutations.

### Adaptive mutations in *MTH1* and *HXT6/7* are selectively mutually exclusive

**Figure 4:**
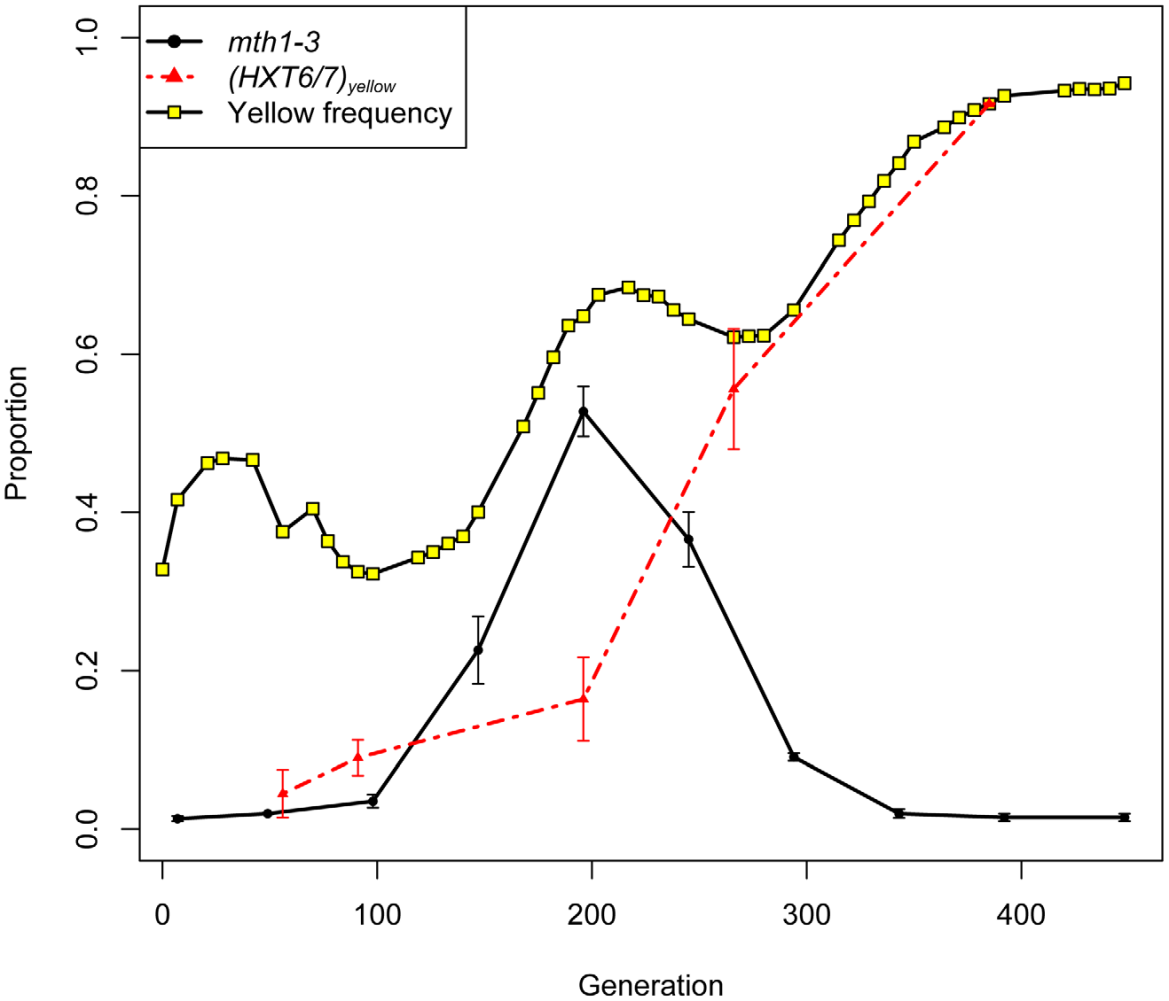
Allele frequencies of *mth1-3* and *HXT6/7 amplification* in the yellow subpopulation. Over the course of the experiment, *mth1-3* transiently increases in frequency but gets outcompeted by clones carrying the *HXT6/7* amplification by the end of the experiment. Error bars are +/− standard error of the mean of three biological replicate experiments. *HXT6/7* data are from [25], plotted as a proportion of the yellow subpopulation.

The within-population reproducibility of adaptation between different lineages was striking - three independent *mth1* adaptive nonsense mutations arose in clones M1–M3 (hereafter referred to as *mth1-1*, *mth1-2* and *mth1-3*), while the amplification of the tandemly arrayed glucose transporter genes *HXT6* and *HXT7* arose independently in clones M4 and M5, as well as within the green subpopulation by the end of the evolution experiment (see Figure 3 in [25]). These repeated independent changes suggest that the presumptive loss of *MTH1* function or increased *HXT6/7* copy number - both of which result in increased *HXT* expression [26], [32] - are effective mechanisms by which yeast can adapt to limiting glucose. Strikingly, despite the common occurrence of these two mutations independently, none of the five clones we characterized had them both. To further investigate this trend, we genotyped 22 randomly picked clones from the yellow subpopulation at generation 266 for the *mth1-3* allele and *HXT6/7* amplification. While the subpopulation was heterogeneous, none of the 22 clones had both mutations (Table S2). Additionally, when examining our estimates of allele frequencies throughout the evolution experiment, we found that the *mth1-3* allele began decreasing in frequency concurrent with the increase in frequency of the *HXT6/7* amplification in the yellow subpopulation, further suggesting that the two mutations did not co-exist within the same clonal lineage (see generations 200–300, Figure 4). We also genotyped 24 random clones isolated from the generation 448 terminal green subpopulation, and again the two mutations were never seen to co-exist, with all 24 clones carrying the *HXT6/7* amplification but not the *mth1-1* allele (Table S2). Since the *MTH1* coding sequence has a large capacity for nonsense mutations (169/434 codons differ by only one nucleotide from stop codons), and since we were only assessing the specific *mth1-1* allele within the terminal green population, we sequenced the full-length *MTH1* coding sequence in 4 of the 24 terminal green subpopulation clones, and found that all four had the wild-type coding sequence (data not shown). Taken together, these results suggest that the *mth1* mutation and *HXT6/7* amplification did not exist together in the same clone to reach detectable frequencies during our experiment, suggesting they may be selectively mutually exclusive.

### Reciprocal sign epistasis for fitness between *mth1* and *HXT6/7* amplification

**Figure 5:**
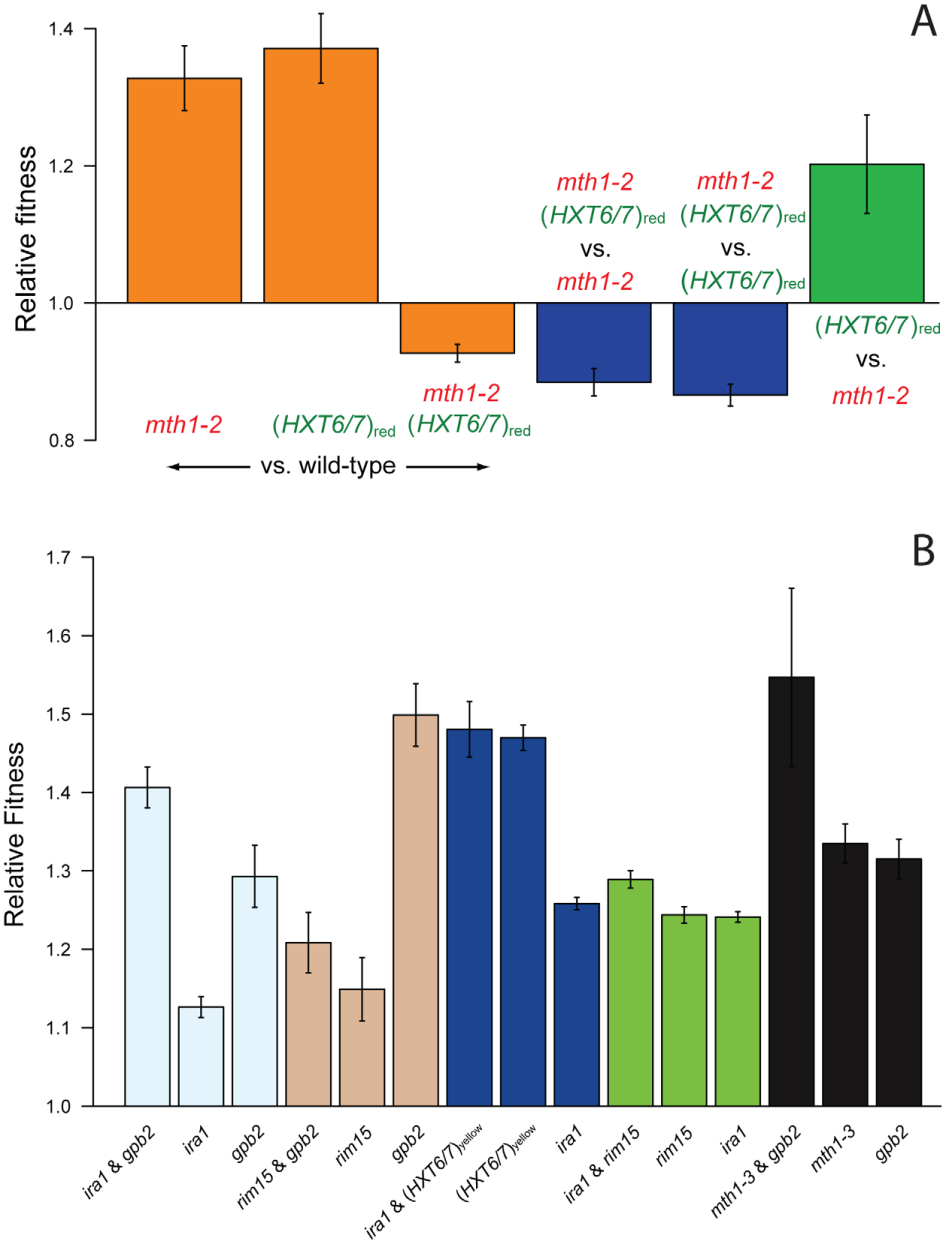
Competition experiments to test for epistasis between singly adaptive mutations. (A) Reciprocal sign epistasis between the *mth1-2* and *HXT6/7* amplification mutations from the red subpopulation, which results in a two-peaked fitness landscape (Figure S5); the *mth1-3* and *HXT6/7* amplification mutations from the yellow subpopulation give similar results (Figure S4). (B) Tests for epistasis between other singly adaptive mutations show pervasive negative epistasis between the pairs *IRA1*/(*HXT6/7*)_yellow_, *IRA1*/*RIM15*, *MTH1*/*GPB2*, as well as sign epistasis between *RIM15* and *GPB2*. Sign epistasis would constrain the fitness landscape but would not lead to two fitness peaks. Error bars are 95% confidence intervals.

Reciprocal sign epistasis for fitness can constrain evolutionary trajectories and even create multiple peaks on a fitness landscape [7], [14], [15], a situation that would create selectively mutually exclusive mutations. Thus, we hypothesized that reciprocal sign epistasis underlies mutual exclusivity between the observed *mth1* mutations and *HXT6/7* amplification. To test this hypothesis, we constructed double mutant strains (as outlined in Figure S3) containing either an *mth1-2* or *mth1-3* nonsense mutation and an *HXT6/7* amplification allele, and competed this double mutant against either a wild-type strain, an *mth1* mutant, or an *HXT6/7* amplification mutant. As controls, we also competed single mutants and wild-type spores from the same dissection against a wild-type strain. As expected, the single mutants were again more fit than the parental wild-type strain. However, the double mutant was significantly less fit than the wild-type strain as well as both the *mth1* and *HXT6/7* amplification single mutant strains (Figure 5A and Figure S4). This shows that within the genetic contexts we tested, a clone with both a nonsense mutation in *mth1* and an amplification in *HXT6/7* is highly maladaptive, and is thus unlikely to reach an appreciable frequency during glucose-limited evolution.

### Pervasive negative epistasis between other adaptive mutations

To determine if sign epistasis during our evolution experiment was a common phenomenon, we constructed pairwise combinations of non-co-existing adaptive mutations from M1–M5 and competed them against the wild-type parental strain. While we saw no further evidence of reciprocal sign epistasis between these pairs of mutations, the *RIM15* and *GPB2* mutations do show standard sign epistasis, where the double mutant is less fit than one of the single mutants (*GPB2*) but more fit than the other (*RIM15*) (Figure 5B). In addition, negative epistasis is also prevalent, defined as the double mutant having a fitness effect that is less than the additive effects of the single mutants, but still greater than the fitness effects of each single mutant. The pairs *IRA1*/(*HXT6/7*)_yellow_, *IRA1*/*RIM15*, *MTH1*/*GPB2* all have significant negative epistasis between the single mutations. Since negative epistasis between adaptive mutations results in a double mutant with a fitness greater than both single mutants, it is selectively favorable and does not act to constrain the fitness landscape [7]. As with the inferred negative epistasis between mutations found in M4 (Figure 3), it is possible that the calculated negative epistasis between these mutations is due to the large magnitude of their individual fitness effects exceeding the maximum fitness, and the seemingly pervasive negative epistasis may be non-specific, possibly occurring between any two mutations of individually large enough fitness effect.

## Discussion

We have characterized the fitness of individual mutations as well as genetic interactions between mutations that arose during experimental evolution of yeast under glucose limitation. Our exhaustive analysis of single mutation fitness determined that only one to two mutations per adaptive clone individually result in a fitness gain, regardless of the total number of mutations in an adaptive clone (Figure 2). The remaining mutations' effects are consistent with the null hypothesis of neutrality. Our data also show that, given an additive model, the identified singly adaptive mutations are sufficient to explain the fitness of adaptive clones M1–M3 and M5, and in one case (M4), there is evidence for negative epistasis between mutations (Figure 3). The effect of negative epistasis may act like the idea of diminishing returns – adaptive mutations of large individual effect result in a smaller fitness effect when they occur together. The idea of diminishing returns of adaptation over time in a constant environment is further supported by data from long-term *E. coli* evolution experiments, where the rate of fitness improvement was initially fast but quickly decreased over time and then remained low [33], [34]. Perhaps this decrease in the adaptive value of accumulated mutations is due to a maximum intrinsic fitness for a particular environment. This makes intuitive sense in the light of the mutations in our study having high relative fitness coefficients (1.1 to 1.45), which when combined under an additive model would result in extremely large fitness coefficients. This non-specific, negative epistasis-like phenomenon is further exemplified by the interactions between non-co-existing adaptive mutations (Figure 5B). Thus, it is possible that the pervasive negative epistasis observed is genetically promiscuous and not a result of the specific interactions between the two mutations, and any two mutations with large enough fitness increases will display such interactions. To our knowledge, this phenomenon has not been addressed in the literature, and there is as yet no distinction between true negative epistasis between specific sets of alleles and a non-specific, negative epistasis-like phenomenon due to a fitness upper bound, even though both scenarios would be classified as negative epistasis by its mathematical definition.

We have also determined that reciprocal sign epistasis between mutations in *MTH1* and *HXT6/7* constrains adaptation, which causes these two mutations to be mutually exclusive during the evolution experiment, a result that is consistent with data from an independent glucose-limited yeast evolution experiment [35]. This constraint can be represented in an empirical fitness landscape, which maps out the mutational evolutionary trajectories available for natural selection, and our data make possible the construction of this landscape using direct fitness effects of combinations of mutations. Reciprocal sign epistasis between the adaptive mutations in *MTH1* and *HXT6/7* can be represented as a simple two-locus fitness landscape (Figure S5). Here, the *mth1* mutation is at a local optimum, as the only mutational steps available lead to a decrease in fitness, and it is not the fittest genotype on the landscape (Figure 5A and Figure S4). The *HXT6/7* amplification mutation is the fittest genotype and is therefore the global optimum for this landscape. A consequence of this landscape is that lineages with an adaptive mutation in *MTH1* are stuck on a local adaptive peak and may not be able to reach the higher fitness peak where the *HXT6/7* amplification mutation lies. This is the likely reason why the *mth1* and *HXT6/7* mutations remain mutually exclusive for the duration the experiment.

The issue of how populations move from one peak to another in nature (the “peak shift” problem) has been disputed since Wright conceived of the fitness landscape metaphor [1], [2], [36]–[38]. Furthermore, the existence of multi-peaked fitness landscapes themselves has recently been questioned at the theoretical level due to their immense multidimensionality causing neutral ridges connecting genotypes of high fitness on the landscape (the holey landscape model) [4], [39]. These ridges thereby eliminate the classical peak shift problem, and there is some experimental support for such ridges (e.g. [21], [40]). Since the adaptive mutations in *mth1* and *HXT6/7* remain mutually exclusive in our experiment, even after sampling several clones in different lineages, this supports the argument of an adaptive valley on the fitness landscape rather than a ridge connecting the two peaks. Alternatively, a ridge connecting the two peaks might be long and circuitous, and our experiment was not performed for sufficient evolutionary time for neutral evolution on a fitness ridge to occur. This is likely the case in [41], where an unresolved potentiating mutation is thought to have occurred 20,000 generations into the evolution experiment, allowing an innovative phenotype to evolve. In either case, the constraint is such that it would be difficult to adapt from one peak to the other.

In reality, all possible genotypes are present on a fitness landscape. In our case, while it is impossible to experimentally test all combinations of all possible mutations in conjunction with the *mth1/(HXT6/7)* double mutant to prove that this portion of the fitness landscape does indeed have two peaks [3], the large population size of the culture (2×10^9^) means that a large spectrum of mutations should be sampled by natural selection often (∼10^7^ new SNP mutations per generation, based on the previously measured mutation rate [29]). This suggests that natural selection may be rejecting the double mutant in myriad genomic contexts. Determining whether further evolution of an *mth1* single mutant strain ever results in an *HXT6/7* amplification mutation will shed additional light on the feasibility of adaptation from one peak to the other. If the *HXT6/7* amplification can indeed appear on the *mth1* background in such an evolution experiment, this would suggest that a compensatory mutation or mutations provide a fitness ridge between the peaks. While observing how an evolution experiment proceeds cannot indisputably prove the shape of the fitness landscape, it can inform the most relevant and repeated paths of adaptation.

It has been understood for quite some time that epistasis is a fundamental component of adaptation [6], but only have recent technological developments facilitated the discovery and testing of individual nucleotide changes and how they interact with one another to create function and fitness [6]–[8], [18]–[22]. We speculate that for the reciprocal sign epistasis between *mth1* and *HXT6/7*, the fitness defect may be caused by an overabundance of hexose transporter proteins in the cell, due to the fact that both individual mutations act to increase hexose transporter transcription [25], [26], [32]. It is possible that the devotion of too many resources to the production of hexose transporters may take resources away from other essential functions. For example, the secretion machinery by which hexose transporters are localized to the plasma membrane may be overwhelmed by their overabundance. Alternatively, the large number of hexose transporters may take up space on the membrane's surface, preventing other transporters from being correctly localized or negatively impacting the fluidity of the membrane. Another scenario is that the overabundant hexose transporters may aggregate and form plaques in the cell due to their 12 hydrophobic trans-membrane domains. Understanding the mechanism of the reciprocal sign epistasis between *mth1* and *HXT6/7* will be important for understanding the underpinnings of molecular fitness landscapes and is worthy of further investigation.

The rugged *mth1*/(*HXT6/7*) fitness landscape has far-reaching evolutionary implications. In the Dobzhansky-Muller model of postzygotic reproductive isolation, two species are separated from each other by a pair of genomic loci that interact negatively to create a hybrid organism that is of lower fitness compared to its parents [42], [43]. This model bears striking similarity to the epistatic interaction we see between *mth1* and *HXT6/7* amplification (also see [23]). In our case, if two lineages became fixed for the *mth1* or *HXT6/7* mutations, the low fitness of hybrid double mutant offspring may lead the two lineages on a path to reproductive isolation, though in contrast to a typical D-M pair, clones containing the individual mutations are more fit than wild-type clones. This is in contrast to a recent finding of a D-M pair between two strains of *S. cerevisiae* that were experimentally adapted to different environmental conditions [9], [44]. In this case, the two mutations were adaptive in the conditions in which they evolved, but maladaptive when made to co-occur in one of the conditions, which is the traditional way in which D-M pairs are thought to arise.

Mutually exclusive mutations are also known to exist in cancers [45], [46]. Of specific relevance is the recent observation that mutually exclusive mutations in the Ras pathway are individually adaptive under limiting glucose in colorectal cells, an environmental condition believed to be relevant to cancers *in vivo* [46]. Intriguingly, these mutations lead to an increased expression of the glucose transporter GLUT1 resulting in increased glucose uptake by cancer cells [46]. These results from human cancers closely parallel the mutations we see in the glucose sensing and Ras/cAMP pathways, which lead to an increase expression of the hexose transporters. Thus, human cancers and yeast may respond to the same selective pressures by mutating the same pathways, and these parallels beg the thought that reciprocal sign epistasis might be the mechanism by which these cancer mutations are mutually exclusive.

## Materials and Methods

### Source of adaptive clones

The details of the experimental setup yielding the adaptive clones used in the present study have been described previously [25]. Briefly, three strains of haploid S288c that are isogenic, except that each expresses a different fluorescent protein constitutively (GFP, YFP or DsRed), were seeded in equal quantities in a 20 ml chemostat device. The population was evolved for 448 generations at steady state under glucose limitation (0.08%) at a dilution rate of 0.2 h^−1^. During this evolution, the proportions of the three colored lineages were tracked using flow cytometry. At five points throughout the evolution experiment, the population was sorted into its component colored subpopulations using fluorescence activated cell sorting (FACS), and 7 clones from the visibly adaptive subpopulation were isolated, competed to determine the clones' fitnesses, and the most fit clone was selected for subsequent investigations. The five resulting clones are labeled M1–M5 (Table S3 and Figure 1).

### Strains and growth conditions

Strains used and constructed in this study are shown in Table S3. All batch and competitive chemostat cultures were grown as described previously [25].

### Sequencing and mutation determination

Adaptive clones M1–M5 (GSY1171, GSY1180, GSY1194, GSY1200, GSY1208) and an ancestral strain (GSY1135) were single-end sequenced using the Illumina Genome Analyzer (GAI or GAII). Single-end sequencing libraries were constructed for each clone using the Illumina Genomic DNA sample prep kit from 5 µg of genomic DNA and each library was sequenced on 2 flow cell lanes. Sequence analysis was performed as follows, using default parameters unless otherwise noted. Reads were mapped to the S288c reference genome (downloaded from SGD on Dec 3, 2008) using bwa-short in BWA v0.5.7 [47] and variants were found with SAMtools v0.1.7-6 [48] and filtered with SAMtools varFilter (-d 5; -D 100,000; -S 20; -i 50). For each genomic position, if the ancestral strain and adaptive clone shared the consensus genotype, the variant was eliminated. The resulting variants were filtered unless they passed the following heuristic filters. SNP: proportion of “N” bases covering position <0.1; majority non-reference base must be >80% of all non-reference bases at the position; when comparing ancestral to evolved, the proportion of non-reference bases must be <0.1 in one strain and >0.5 in the other. Indels: coverage >10; indel calls must make up >50% of coverage; proportion of the sum of two most frequent indel calls >0.8; proportion of reference matches <0.5; when comparing ancestral to evolved, the difference in proportion for shared alleles must be >0.3. All novel SNPs were confirmed by Sanger sequencing (primers in Table S4).

Three SNPs were excluded from further consideration because they were present in both the ancestral strain and each evolved strain of a particular color: chr02:353579 (C to A) in GSY1136 and M1; chr09:275382 (*PKP1*, G to T) in GSY1135, M2 and M4; and chr16:401704 (*MOT1*, G to C) in GSY1137, M3 and M5. In addition, the *COX18* mutation found in M2 and M4 as reported in [25] was also excluded, because it was determined the mutation was already fixed in the red subpopulation at the earliest sampling. Thus the mutation was not the result of evolution in the chemostat but was most likely a random mutation in the colony used to initiate the red population in the chemostat; we have also determined that this mutation is not adaptive (data not shown).

### Assessing the copy number of the *HXT6/7* array

Real-time quantitative PCR (qPCR) of genomic DNA (gDNA) was used to determine the copy number of the *HXT6/7* coding region, as well as the *HXT7* promoter, using a previously described protocol [49] and primers listed in Table S4. Results were normalized to the copy number of *UBP1*, a non-varying locus also on chromosome 4, to control for slight differences in input gDNA amount. Experiments were performed in triplicate. 95% confidence intervals were calculated as mean ± 1.96 * SEM.

*HXT6/7* copy number was also visualized using sequencing coverage. Adaptive clone coverage was normalized to the average coverage of the ancestral strain and this was divided by the ancestral strain coverage. A running median was calculated over this proportion to smooth the data.

### Construction of strains for competition experiments

M1–M5 were backcrossed to a wild-type S288c ancestral strain containing the same fluorescent protein (GSY1221–1223). Diploids were sporulated and dissected, and resulting spores were genotyped for mutations using allele-specific colony PCR (primers in Table S4). The spores were also tested for mating type by cross-stamping two tester strains (GSY2476 and GSY2670) onto YPD master plates of the dissections, grown overnight, followed by replica plating and selection for mated diploids on SC-ura+G418 plates. Backcrossing was repeated as often as necessary to get a single mutation segregating per cross (Figure S6). Double mutants for epistasis experiments were constructed by mating haploid single mutant strains and the resulting diploids were sporulated, dissected and genotyped.

### Pairwise competition experiments

Pairwise competitive chemostats were performed as described [25], but were sampled every 6 h over 20–25 generations. Single mutation competition experiments were performed in at least biological triplicate. As a control, we also performed competition experiments in at least biological triplicate of wild-type sister spores derived from the same backcross. Selection coefficients were calculated as described [25], and normalized by subtracting the wild-type mean selection coefficient from the mutant mean selection coefficient for each mutation. Fitness was calculated relative to the competing wild-type strain, such that 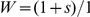. The *mth1*/(*HXT6/7*) epistasis competitive chemostats were performed in at least biological duplicate, and the remaining epistasis competitive chemostats were performed once. For each of these experiments, a wild-type strain from the same dissection was used as a control. The linear phase of growth was determined, and the selection coefficient was taken as the slope of the linear regression line as described [35] and normalized as above. 95% confidence intervals were inferred for the slope of the regression using the confint() function in R. Analysis of covariance (ANCOVA) was used to determine if the selection coefficient of the mutant strain was significantly different than the wild-type control. In this analysis, a model in which the mutant and wild-type experiments were allowed independent slopes was compared to a model in which they had a common slope. Epistasis was quantified as 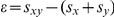, where *s* is the normalized selection coefficient and *x* and *y* are mutant alleles of two different genes [6]. Error of epsilon was calculated by the method of error propagation: 
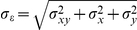
 [22], and a 95% confidence interval of epsilon was calculated as 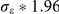. If the value of epsilon fell outside this confidence interval, we considered there to be significant epistasis between alleles *x* and *y*.

### Assessing the additive effects of individual adaptive mutations

For each adaptive clone, the selection coefficients of all individually adaptive mutations derived from that clone were added and a pseudo relative fitness was calculated from the selection coefficient as above. This was compared to the relative fitness data for each clone from [25]. To make this comparison quantitatively, we used the epistasis framework above to calculate a value and confidence interval for epsilon. If indeed there are additional adaptive mutations, this would manifest itself as a positive value for epsilon, as the additive effects of the individual mutations would be less than the fitness of the clone.

### Population allele frequencies

Quantitative Sanger sequencing [35] and the software PeakPicker [50] with default settings were used to determine the frequency of *mth1-3* in triplicate throughout the evolution.

### Data availability

The Illumina sequence data are available at the NCBI Sequence Read Archive under accession SRA020606.1.

## Supporting Information

Figure S1 Relative copy number of the *HXT6/7* amplification determined by sequencing coverage. (A) M4 (B) M5. Data are compared to an ancestral strain to show relative copy number. Blue line through data is a running median. Diagram shows *HXT6* and HXT7 coding regions, as well as nearby genes.
(TIF)

Figure S2 Relative copy number of the *HXT6/7* coding regions and *HXT7* promoter deter- mined by real-time quantitative PCR (qPCR). The *HXT6/7* coding primer targets both *HXT6* and *HXT7*, which flank the *HXT7* promoter. The qPCR results of the adaptive clones were compared to an ancestral strain without the amplification, which has one copy each of *HXT6* and *HXT7*, and one copy of the *HXT7* promoter. Error bars are 95% confidence intervals.
(TIF)

Figure S3 Experimental setup for testing for the presence of epistasis between adaptive mutations. Single mutants of opposite mating types were crossed, sporulated, dissected and genotyped, yielding the four possible genotypic classes of spores. These spores were then competed against a wild-type strain to determine the fitness effect of each combination of mutations. Wild-type versus wild-type competitions were included as internal controls and data were normalized to these experiments. *mth1* and *HXT6/7* are used here as examples.
(TIF)

Figure S4 Competition experiments to test for epistasis between *mth1-3* and (*HXT6/7*)_yellow_. Results show reciprocal sign epistasis between the *mth1-3* and *HXT6/7* amplification mutations from the yellow subpopulation. (*HXT6/7*)_y_ = (*HXT6/7*)_yellow_.
(TIF)

Figure S5 Empirical fitness landscape describing reciprocal sign epistasis between *mth1* and the *HXT6/7* amplification. The vertical z-axis shows relative fitness from Figure 4 (bars 1–3), with the wild-type genotype residing on the plane of fitness equal to one. This reciprocal sign epistasis leads to two fitness peaks, located at each single mutant. The double mutant has fitness lower than the wild-type, forcing the fitness planes to slice through the horizontal plane describing a relative fitness of one. A two-peaked fitness landscape is significant because an individual at a local optimum (*mth1*) cannot reach the global optimum (*HXT6/7*) without traversing a fitness valley, which is strongly disfavored by natural selection alone.
(TIF)

Figure S6 Experimental setup for testing the fitness effect of each mutation. Each adaptive clone was backcrossed until each individual mutation was segregating 2∶2 per yeast tetrad. Com- petitive chemostats were then performed against a wild-type strain for the single mutation spores and wild-type spores as internal controls.
(TIF)

Table S1 Summary of Illumina sequencing statistics of the ancestral strain and M1–M5.
(DOC)

Table S2 Genotyping results for mutations in *MTH1* and *HXT6/7* from random clones isolated from the indicated generation and colored subpopulation. Mutant alleles are in bold underline. The generation 266 yellow subpopulation in heterogeneous, containing both *mth1* and *HXT6/7* amplification mutations, but none of the random clones genotyped carry both mutations. The generation 448 green subpopulation is homogeneous for the *HXT6/7* amplification. Mutations in *MTH1* and *HXT6/7* never co-occur, suggesting that mutations in these two genes are selectively mutually exclusive.
(DOC)

Table S3 Strain list.
(DOC)

Table S4 Primers used. Usage key: A – allele-specific PCR, B – mutation confirmation by Sanger sequencing, C – quantitative Sanger sequencing, D – quantitative PCR.
(DOC)
