# Supplementary figures and images for: Reciprocal Sign Epistasis between Frequently Experimentally Evolved Adaptive Mutations Causes a Rugged Fitness Landscape
Source: PLoS Genet. 2011 Apr 28;7(4):e1002056. doi: 10.1371/journal.pgen.1002056 (PMC3084205; doi:10.1371/journal.pgen.1002056)

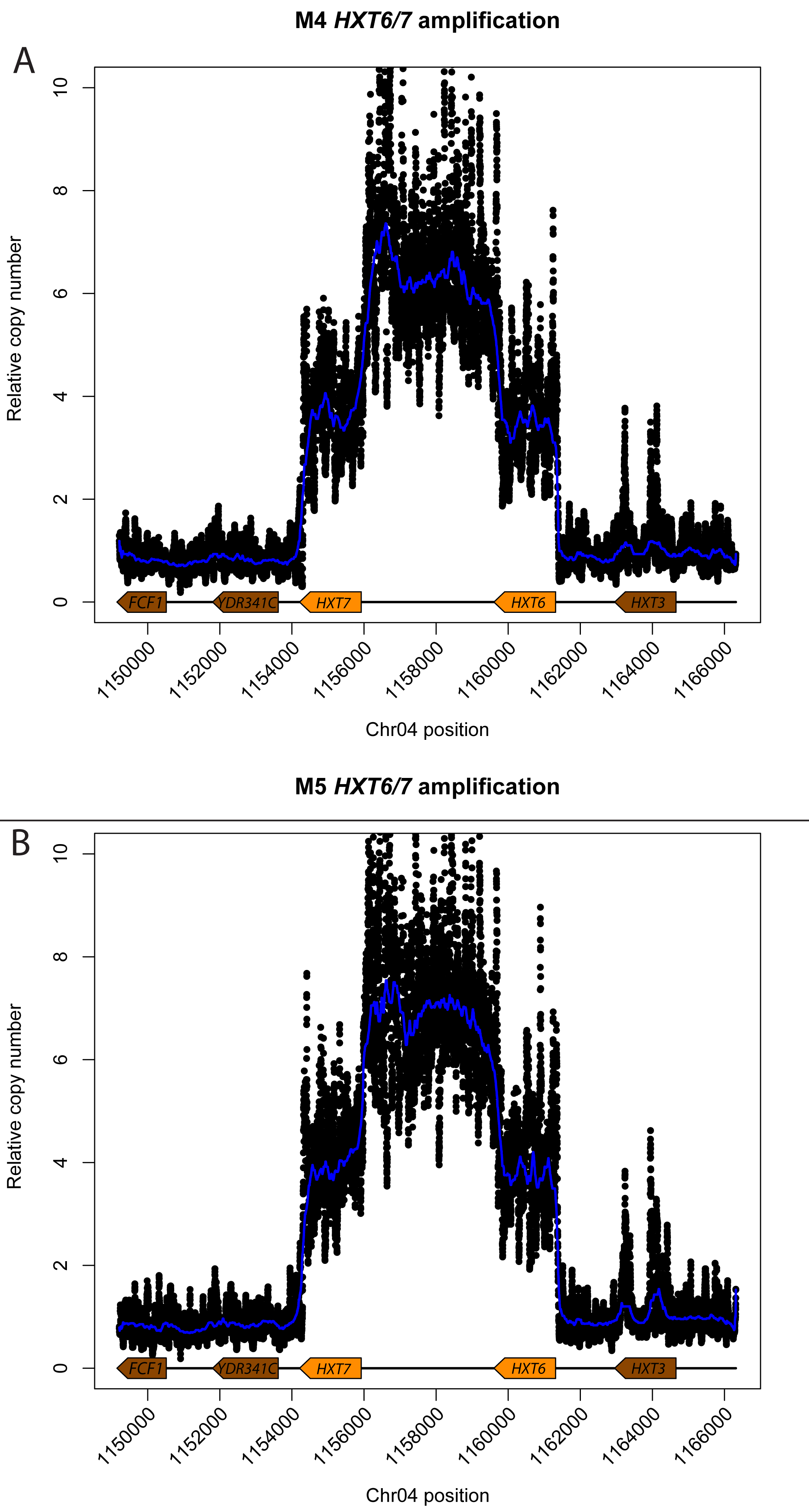

Supplement: Figure S1 — Relative copy number of the HXT6/7 amplification determined by sequencing coverage. (A) M4 (B) M5. Data are compared to an ancestral strain to show relative copy number. Blue line through data is a running median. Diagram shows HXT6 and HXT7 coding regions, as well as nearby genes. (TIF) [file pgen.1002056.s001.tif]

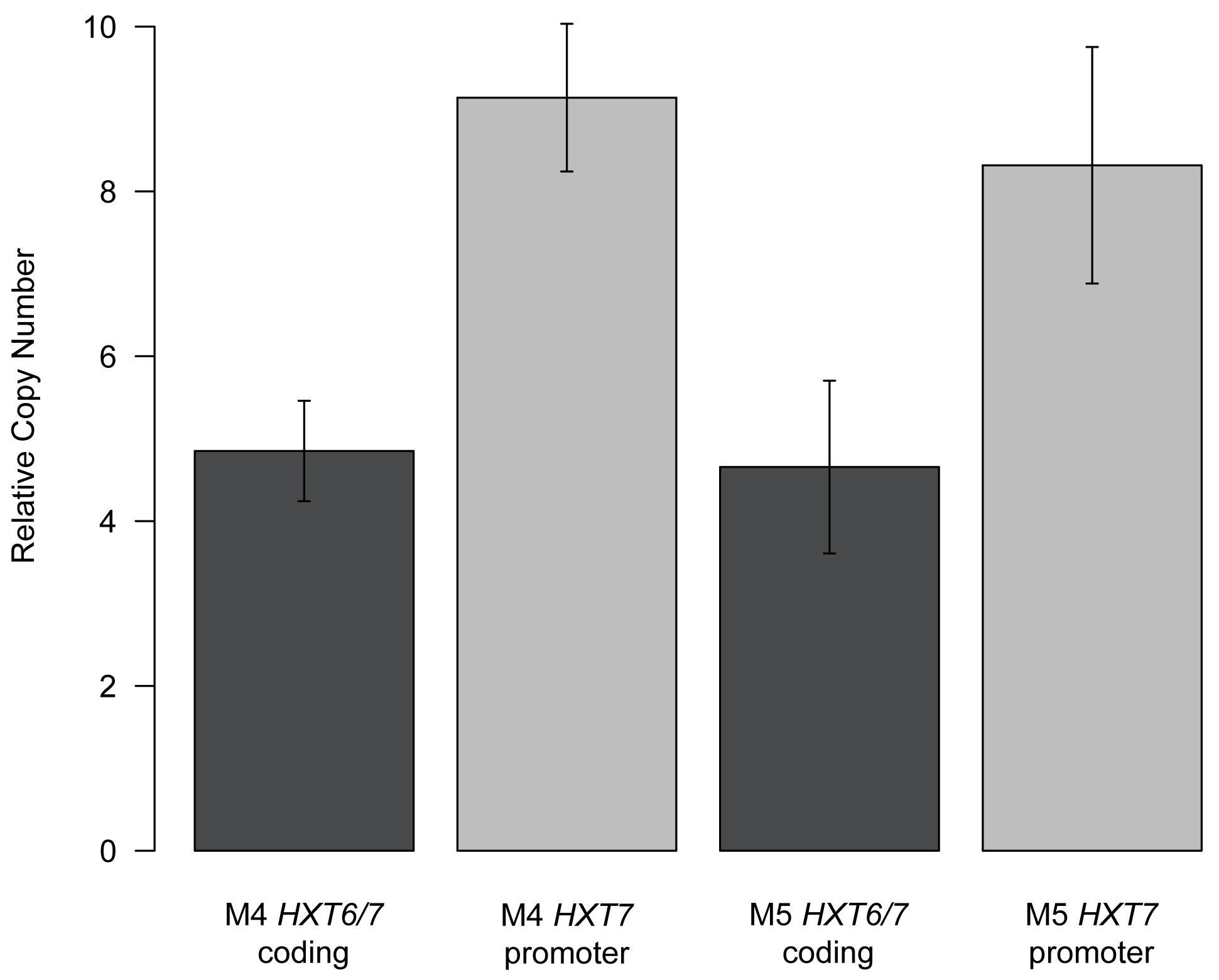

Supplement: Figure S2 — Relative copy number of the HXT6/7 coding regions and HXT7 promoter deter- mined by real-time quantitative PCR (qPCR). The HXT6/7 coding primer targets both HXT6 and HXT7, which flank the HXT7 promoter. The qPCR results of the adaptive clones were compared to an ancestral strain without the amplification, which has one copy each of HXT6 and HXT7, and one copy of the HXT7 promoter. Error bars are 95% confidence intervals. (TIF) [file pgen.1002056.s002.tif]

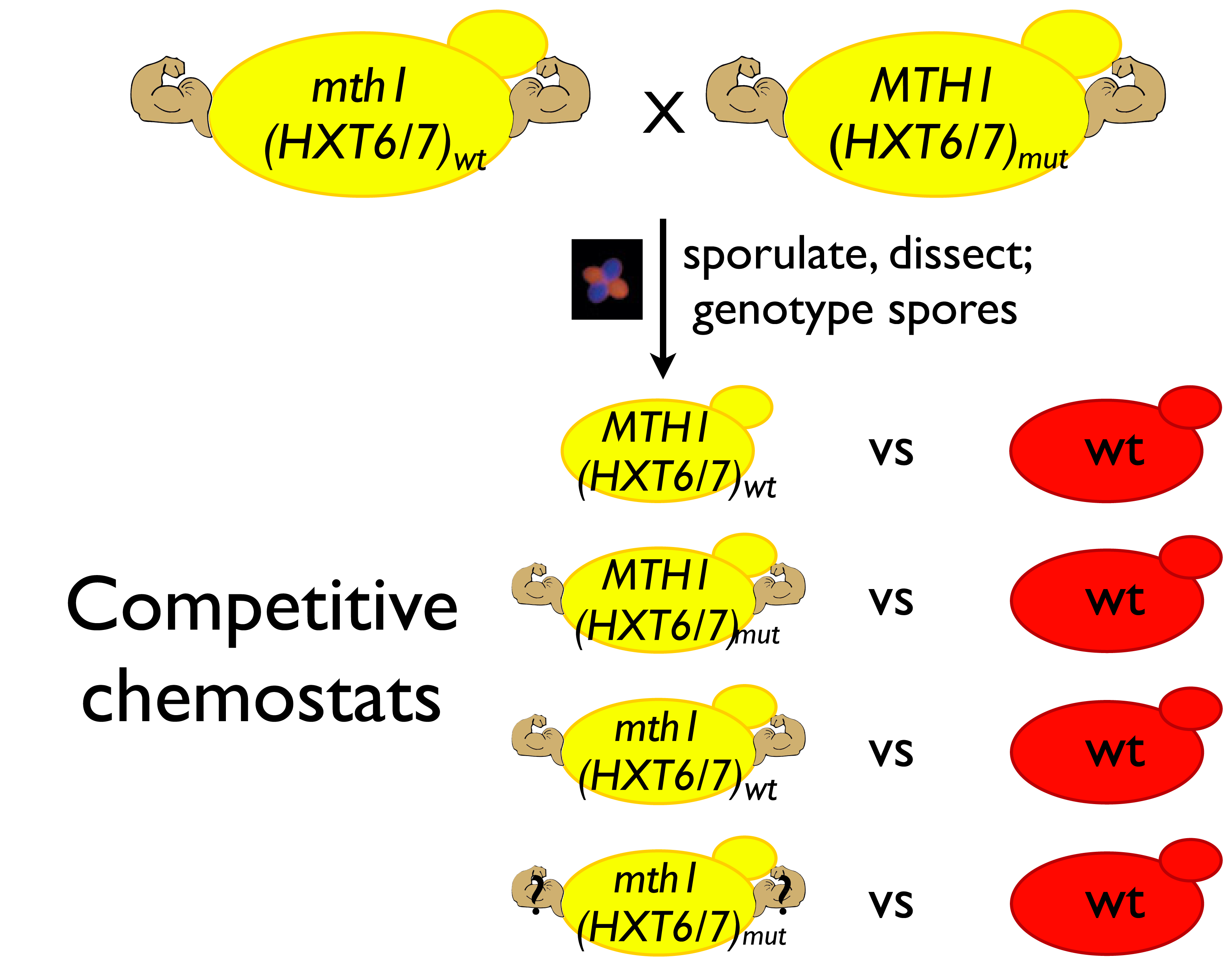

Supplement: Figure S3 — Experimental setup for testing for the presence of epistasis between adaptive mutations. Single mutants of opposite mating types were crossed, sporulated, dissected and genotyped, yielding the four possible genotypic classes of spores. These spores were then competed against a wild-type strain to determine the fitness effect of each combination of mutations. Wild-type versus wild-type competitions were included as internal controls and data were normalized to these experiments. mth1 and HXT6/7 are used here as examples. (TIF) [file pgen.1002056.s003.tif]

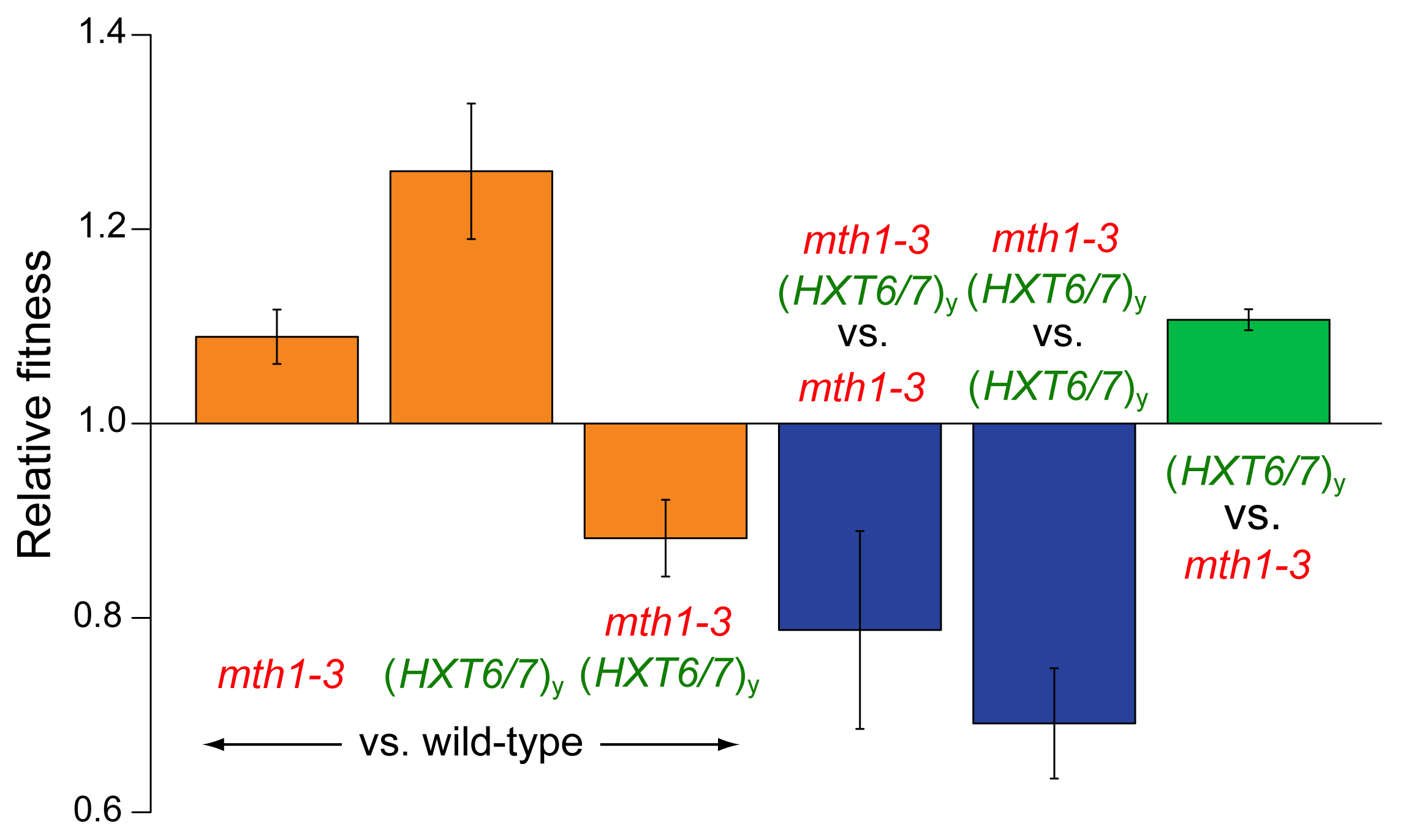

Supplement: Figure S4 — Competition experiments to test for epistasis between mth1-3 and (HXT6/7)yellow. Results show reciprocal sign epistasis between the mth1-3 and HXT6/7 amplification mutations from the yellow subpopulation. (HXT6/7)y = (HXT6/7)yellow. (TIF) [file pgen.1002056.s004.tif]

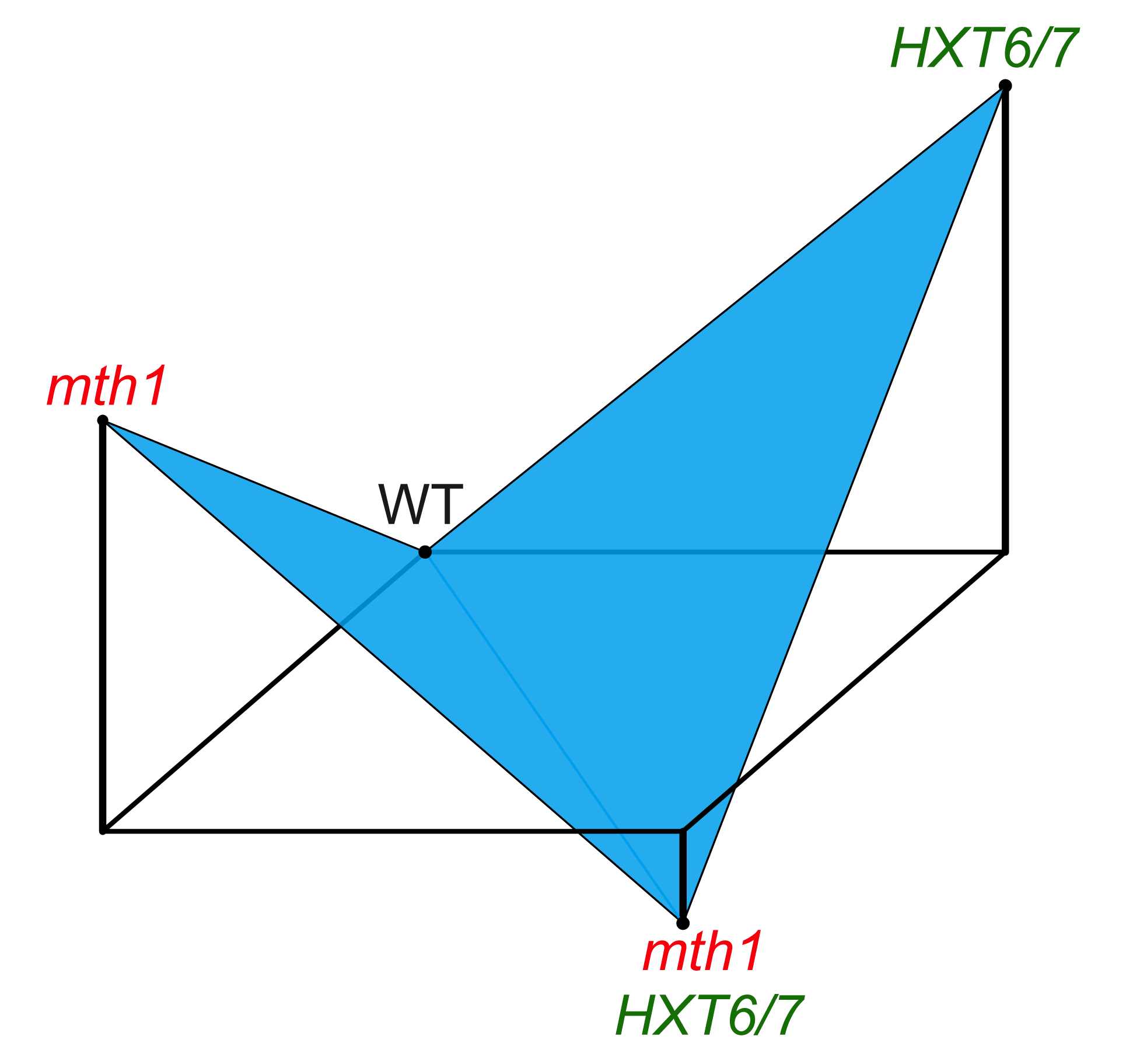

Supplement: Figure S5 — Empirical fitness landscape describing reciprocal sign epistasis between mth1 and the HXT6/7 amplification. The vertical z-axis shows relative fitness from Figure 4 (bars 1–3), with the wild-type genotype residing on the plane of fitness equal to one. This reciprocal sign epistasis leads to two fitness peaks, located at each single mutant. The double mutant has fitness lower than the wild-type, forcing the fitness planes to slice through the horizontal plane describing a relative fitness of one. A two-peaked fitness landscape is significant because an individual at a local optimum (mth1) cannot reach the global optimum (HXT6/7) without traversing a fitness valley, which is strongly disfavored by natural selection alone. (TIF) [file pgen.1002056.s005.tif]

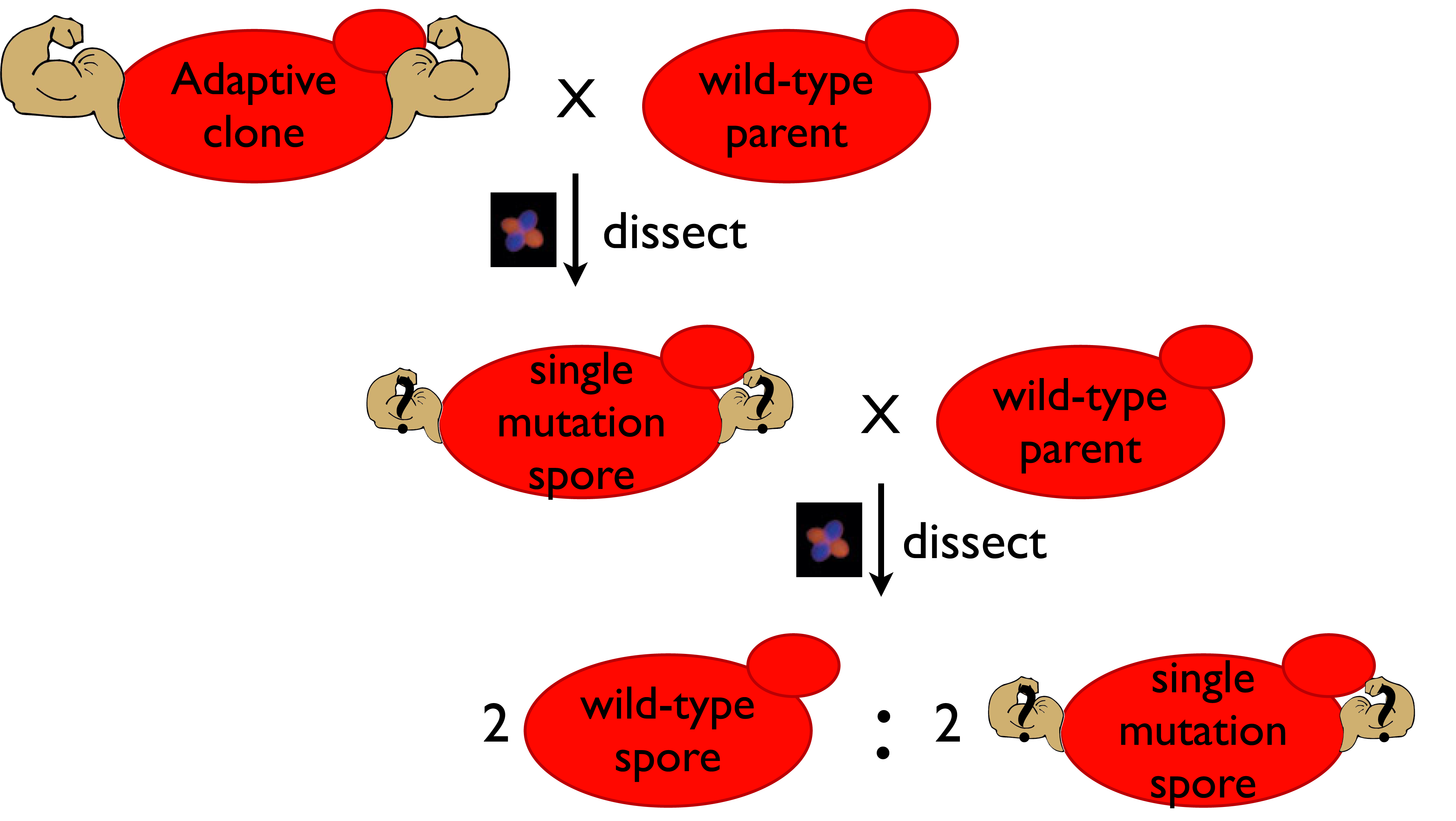

Supplement: Figure S6 — Experimental setup for testing the fitness effect of each mutation. Each adaptive clone was backcrossed until each individual mutation was segregating 2∶2 per yeast tetrad. Com- petitive chemostats were then performed against a wild-type strain for the single mutation spores and wild-type spores as internal controls. (TIF) [file pgen.1002056.s006.tif]
