# Supplementary material for: Reciprocal Sign Epistasis between Frequently Experimentally Evolved Adaptive Mutations Causes a Rugged Fitness Landscape
Source: PLoS Genet. 2011 Apr 28;7(4):e1002056. doi: 10.1371/journal.pgen.1002056 (PMC3084205; doi:10.1371/journal.pgen.1002056)
Supplement: Table S1 — Summary of Illumina sequencing statistics of the ancestral strain and M1–M5. (DOC) [file pgen.1002056.s007.doc]

|  | **Ancestor** | **M1** | **M2** | **M3** | **M4** | **M5** |
| --- | --- | --- | --- | --- | --- | --- |
| **Total reads** | 18,984,170 | 9,361,251 | 13,793,258 | 24,034,054 | 18,787,801 | 17,041,684 |
| **Mapped reads** | 11,985,828  (63.1%) | 7,739,640  (82.7%) | 10,249,286  (74.3%) | 16,317,342  (67.9%) | 12,487,454  (66.5%) | 10,615,765  (62.3%) |
| **Nuclear genome coverage** | 32.6X | 21.5X | 27.5X | 45.8X | 34.9X | 29.4X |
| **Mitochondrial coverage** | 443X | 227X | 429X | 403X | 328X | 313X |
