# Supplementary material for: Reciprocal Sign Epistasis between Frequently Experimentally Evolved Adaptive Mutations Causes a Rugged Fitness Landscape
Source: PLoS Genet. 2011 Apr 28;7(4):e1002056. doi: 10.1371/journal.pgen.1002056 (PMC3084205; doi:10.1371/journal.pgen.1002056)
Supplement: Table S2 — Genotyping results for mutations in MTH1 and HXT6/7 from random clones isolated from the indicated generation and colored subpopulation. Mutant alleles are in bold underline. The generation 266 yellow subpopulation in heterogeneous, containing both mth1 and HXT6/7 amplification mutations, but none of the random clones genotyped carry both mutations. The generation 448 green subpopulation is homogeneous for the HXT6/7 amplification. Mutations in MTH1 and HXT6/7 never co-occur, suggesting that mutations in these two genes are selectively mutually exclusive. (DOC) [file pgen.1002056.s008.doc]

| Generation: Subpopulation | Number of clones genotyped | *MTH1*  *HXT6/7* | ***mth1***  *HXT6/7* | *MTH1*  ***HXT6/7*** | ***mth1***  ***HXT6/7*** |
| --- | --- | --- | --- | --- | --- |
| 266: Yellow | 22 | 7 | 12 | 3 | 0 |
| 448: Green | 24 | 0 | 0 | 24 | 0 |
