# Supplementary material for: Reciprocal Sign Epistasis between Frequently Experimentally Evolved Adaptive Mutations Causes a Rugged Fitness Landscape
Source: PLoS Genet. 2011 Apr 28;7(4):e1002056. doi: 10.1371/journal.pgen.1002056 (PMC3084205; doi:10.1371/journal.pgen.1002056)
Supplement: Table S3 — Strain list. (DOC) [file pgen.1002056.s009.doc]

| **Strain** | **Name** | **Genotype** | **Reference** |
| --- | --- | --- | --- |
| **GSY280** | FY3 | MATa ura3-52 | [52] |
| **GSY1135** | DsRed α | MATα ura3-52 YBR209W::pGS64::ybr209w pkp1 | [25] |
| **GSY1136** | GFP α | MATα ura3-52 YBR209W::pGS62::ybr209w chr02:353579 | [25] |
| **GSY1137** | YFP α | MATα ura3-52 YBR209W::pGS63::ybr209w mot1 | [25] |
| **GSY1221** | GFP a | MATa ura3-52 YBR209W::pGS62::ybr209w chr02:353579 | This study; Spore of GSY1136 x GSY280 |
| **GSY1222** | YFP a | MATa ura3-52 YBR209W::pGS63::ybr209w mot1 | This study; Spore of GSY1137 x GSY280 |
| **GSY1223** | DsRed a | MATa ura3-52 YBR209W::pGS64::ybr209w pkp1 | This study; Spore of GSY1135 x GSY280 |
| **GSY1171** | M1 (C1-7g4) | MATα ura3-52 YBR209W::pGS63::ybr209w mth1-1 chr02:353579 | [25] |
| **GSY1180** | M2 (C1-11r6) | MATα ura3-52 YBR209W::pGS62::ybr209w mth1-2 cox18 chr15:301008 pkp1 | [25] |
| **GSY1194** | M3 (C1-22y5) | MATα ura3-52 YBR209W::pGS64::ybr209w mth1-3 ira1 mot1 | [25] |
| **GSY1200** | M4 (C1-29r4) | MATα ura3-52 YBR209W::pGS62::ybr209w taf5 (HXT6/7)_red_ rim15 cox18 mnn4 chr16:912523 pkp1 | [25] |
| **GSY1208** | M5 (C1-42y5) | MATα ura3-52 YBR209W::pGS64::ybr209w gpb2 (HXT6/7)_yellow_ vma8 dal81 bye1 sly41 muk1 mot1 | [25] |
| **GSY2476** | JWY49 | MATa met15 ura3 leu2 lys2 his3 xks1Δ::KanMX | [51] |
| **GSY2670** | JWY51 | MATα ura3 leu2 his3 xks1::KanMX | J. Wenger |
| **GSY2671** | C1-42y5_2c1d1a | MATα ura3-52 YBR209W::pGS64::ybr209w mot1 | This study |
| **GSY2672** | C1-42y5_2c1d1b | MATa ura3-52 YBR209W::pGS64::ybr209w bye1 mot1 | This study |
| **GSY2673** | C1-42y5_2c1d1c | MATa ura3-52 YBR209W::pGS64::ybr209w mot1 | This study |
| **GSY2674** | C1-42y5_2c1d1d | MATα ura3-52 YBR209W::pGS64::ybr209w bye1 mot1 | This study |
| **GSY2675** | C1-42y5_2c1d2a | MATa ura3-52 YBR209W::pGS64::ybr209w mot1 | This study |
| **GSY2676** | C1-42y5_2c1d2b | MATα ura3-52 YBR209W::pGS64::ybr209w bye1 mot1 | This study |
| **GSY2677** | C1-42y5_2c1d2c | MATa ura3-52 YBR209W::pGS64::ybr209w mot1 | This study |
| **GSY2678** | C1-42y5_2c1d2d | MATα ura3-52 YBR209W::pGS64::ybr209w bye1 mot1 | This study |
| **GSY2679** | C1-29r4_9a2c1a | MATα ura3-52 YBR209W::pGS62::ybr209w chr16:912523 pkp1 | This study |
| **GSY2680** | C1-29r4_9a2c1b | MATα ura3-52 YBR209W::pGS62::ybr209w pkp1 | This study |
| **GSY2681** | C1-29r4_9a2c1c | MATa ura3-52 YBR209W::pGS62::ybr209w pkp1 | This study |
| **GSY2682** | C1-29r4_9a2c1d | MATa ura3-52 YBR209W::pGS62::ybr209w chr16:912523 pkp1 | This study |
| **GSY2683** | C1-29r4_9a2c2a | MATa ura3-52 YBR209W::pGS62::ybr209w chr16:912523 pkp1 | This study |
| **GSY2684** | C1-29r4_9a2c2b | MATa ura3-52 YBR209W::pGS62::ybr209w pkp1 | This study |
| **GSY2685** | C1-29r4_9a2c2c | MATα ura3-52 YBR209W::pGS62::ybr209w chr16:912523 pkp1 | This study |
| **GSY2686** | C1-29r4_9a2c2d | MATα ura3-52 YBR209W::pGS62::ybr209w pkp1 | This study |
| **GSY2687** | C1-42y5_7d2a1a | MATα ura3-52 YBR209W::pGS64::ybr209w mot1 | This study |
| **GSY2688** | C1-42y5_7d2a1b | MATα ura3-52 YBR209W::pGS64::ybr209w mot1 | This study |
| **GSY2689** | C1-42y5_7d2a1c | MATa ura3-52 YBR209W::pGS64::ybr209w dal81 mot1 | This study |
| **GSY2690** | C1-42y5_7d2a1d | MATa ura3-52 YBR209W::pGS64::ybr209w dal81 mot1 | This study |
| **GSY2691** | C1-42y5_7d2a2b | MATa ura3-52 YBR209W::pGS64::ybr209w dal81 mot1 | This study |
| **GSY2692** | C1-42y5_7d2a2c | MATa ura3-52 YBR209W::pGS64::ybr209w dal81 mot1 | This study |
| **GSY2693** | C1-42y5_7d2a2d | MATα ura3-52 YBR209W::pGS64::ybr209w mot1 | This study |
| **GSY2694** | C1-42y5_1b2c1a | MATα ura3-52 YBR209W::pGS64::ybr209w mot1 | This study |
| **GSY2695** | C1-42y5_1b2c1b | MATα ura3-52 YBR209W::pGS64::ybr209w gpb2 mot1 | This study |
| **GSY2696** | C1-42y5_1b2c1c | MATa ura3-52 YBR209W::pGS64::ybr209w mot1 | This study |
| **GSY2697** | C1-42y5_1b2c2a | MATa ura3-52 YBR209W::pGS64::ybr209w gpb2 mot1 | This study |
| **GSY2698** | C1-42y5_1b2c2b | MATα ura3-52 YBR209W::pGS64::ybr209w mot1 | This study |
| **GSY2699** | C1-42y5_1b2c2c | MATα ura3-52 YBR209W::pGS64::ybr209w mot1 | This study |
| **GSY2700** | C1-42y5_1b2c2d | MATa ura3-52 YBR209W::pGS64::ybr209w gpb2 mot1 | This study |
| **GSY2701** | C1-29r4_11c1a | MATa ura3-52 YBR209W::pGS62::ybr209w (HXT6/7)_red_ pkp1 | This study |
| **GSY2702** | C1-29r4_11c1c | MATα ura3-52 YBR209W::pGS62::ybr209w pkp1 | This study |
| **GSY2703** | C1-29r4_11c1d | MATα ura3-52 YBR209W::pGS62::ybr209w (HXT6/7)_red_ pkp1 | This study |
| **GSY2704** | C1-29r4_11c2a | MATa ura3-52 YBR209W::pGS62::ybr209w pkp1 | This study |
| **GSY2705** | C1-29r4_11c2c | MATa ura3-52 YBR209W::pGS62::ybr209w pkp1 | This study |
| **GSY2706** | C1-29r4_11c2d | MATα ura3-52 YBR209W::pGS62::ybr209w (HXT6/7)_red_ pkp1 | This study |
| **GSY2707** | C1-42y5_2c1c1a | MATα ura3-52 YBR209W::pGS64::ybr209w (HXT6/7)_yellow_ mot1 | This study |
| **GSY2708** | C1-42y5_2c1c1b | MATa ura3-52 YBR209W::pGS64::ybr209w (HXT6/7)_yellow_ mot1 | This study |
| **GSY2709** | C1-42y5_2c1c1c | MATa ura3-52 YBR209W::pGS64::ybr209w mot1 | This study |
| **GSY2710** | C1-42y5_2c1c1d | MATα ura3-52 YBR209W::pGS64::ybr209w mot1 | This study |
| **GSY2711** | C1-42y5_2c1c2a | MATα ura3-52 YBR209W::pGS64::ybr209w (HXT6/7)_yellow_ mot1 | This study |
| **GSY2712** | C1-42y5_2c1c2b | MATα ura3-52 YBR209W::pGS64::ybr209w (HXT6/7)_yellow_ mot1 | This study |
| **GSY2713** | C1-42y5_2c1c2c | MATa ura3-52 YBR209W::pGS64::ybr209w mot1 | This study |
| **GSY2714** | C1-42y5_2c1c2d | MATa ura3-52 YBR209W::pGS64::ybr209w mot1 | This study |
| **GSY2715** | C1-22y5_1b1a | MATa ura3-52 YBR209W::pGS64::ybr209w ira1 mot1 | This study |
| **GSY2716** | C1-22y5_1b1b | MATα ura3-52 YBR209W::pGS64::ybr209w mot1 | This study |
| **GSY2717** | C1-22y5_1b1c | MATa ura3-52 YBR209W::pGS64::ybr209w mot1 | This study |
| **GSY2718** | C1-22y5_1b1d | MATα ura3-52 YBR209W::pGS64::ybr209w ira1 mot1 | This study |
| **GSY2719** | C1-22y5_1b2a | MATa ura3-52 YBR209W::pGS64::ybr209w ira1 mot1 | This study |
| **GSY2720** | C1-22y5_1b2b | MATa ura3-52 YBR209W::pGS64::ybr209w ira1 mot1 | This study |
| **GSY2721** | C1-22y5_1b2c | MATα ura3-52 YBR209W::pGS64::ybr209w mot1 | This study |
| **GSY2722** | C1-22y5_1b2d | MATα ura3-52 YBR209W::pGS64::ybr209w mot1 | This study |
| **GSY2723** | C1-11r6_1a1a | MATα ura3-52 YBR209W::pGS62::ybr209w pkp1 | This study |
| **GSY2724** | C1-11r6_1a1b | MATα ura3-52 YBR209W::pGS62::ybr209w chr15:301008 pkp1 | This study |
| **GSY2725** | C1-11r6_1a1c | MATa ura3-52 YBR209W::pGS62::ybr209w chr15:301008 pkp1 | This study |
| **GSY2726** | C1-11r6_1a1d | MATa ura3-52 YBR209W::pGS62::ybr209w pkp1 | This study |
| **GSY2727** | C1-11r6_1a2a | MATa ura3-52 YBR209W::pGS62::ybr209w chr15:301008 pkp1 | This study |
| **GSY2728** | C1-11r6_1a2b | MATa ura3-52 YBR209W::pGS62::ybr209w chr15:301008 pkp1 | This study |
| **GSY2729** | C1-11r6_1a2c | MATα ura3-52 YBR209W::pGS62::ybr209w pkp1 | This study |
| **GSY2730** | C1-11r6_1a2d | MATα ura3-52 YBR209W::pGS62::ybr209w pkp1 | This study |
| **GSY2731** | C1-11r6_1a3a | MATα ura3-52 YBR209W::pGS62::ybr209w chr15:301008 pkp1 | This study |
| **GSY2732** | C1-11r6_1a3b | MATa ura3-52 YBR209W::pGS62::ybr209w pkp1 | This study |
| **GSY2733** | C1-11r6_1a3c | MATα ura3-52 YBR209W::pGS62::ybr209w pkp1 | This study |
| **GSY2734** | C1-11r6_1a3d | MATa ura3-52 YBR209W::pGS62::ybr209w chr15:301008 pkp1 | This study |
| **GSY2735** | C1-29r4_5a1d1a | MATα ura3-52 YBR209W::pGS62::ybr209w pkp1 | This study |
| **GSY2736** | C1-29r4_5a1d1b | MATa ura3-52 YBR209W::pGS62::ybr209w mnn4 pkp1 | This study |
| **GSY2737** | C1-29r4_5a1d1c | MATa ura3-52 YBR209W::pGS62::ybr209w pkp1 | This study |
| **GSY2738** | C1-29r4_5a1d1d | MATα ura3-52 YBR209W::pGS62::ybr209w mnn4 pkp1 | This study |
| **GSY2739** | C1-29r4_5a1d2a | MATα ura3-52 YBR209W::pGS62::ybr209w mnn4 pkp1 | This study |
| **GSY2740** | C1-29r4_5a1d2c | MATα ura3-52 YBR209W::pGS62::ybr209w mnn4 pkp1 | This study |
| **GSY2741** | C1-29r4_5a1d2d | MATa ura3-52 YBR209W::pGS62::ybr209w pkp1 | This study |
| **GSY2742** | C1-7g4_1a | MATa ura3-52 YBR209W::pGS62::ybr209w mth1-1 chr02:353579 | This study |
| **GSY2743** | C1-7g4_1b | MATα ura3-52 YBR209W::pGS62::ybr209w chr02:353579 | This study |
| **GSY2744** | C1-7g4_1c | MATa ura3-52 YBR209W::pGS62::ybr209w chr02:353579 | This study |
| **GSY2745** | C1-7g4_1d | MATα ura3-52 YBR209W::pGS62::ybr209w mth1-1 chr02:353579 | This study |
| **GSY2746** | C1-7g4_2a | MATα ura3-52 YBR209W::pGS62::ybr209w chr02:353579 | This study |
| **GSY2747** | C1-7g4_2b | MATa ura3-52 YBR209W::pGS62::ybr209w mth1-1 chr02:353579 | This study |
| **GSY2748** | C1-7g4_2c | MATα ura3-52 YBR209W::pGS62::ybr209w mth1-1 chr02:353579 | This study |
| **GSY2749** | C1-7g4_2d | MATa ura3-52 YBR209W::pGS62::ybr209w chr02:353579 | This study |
| **GSY2750** | C1-11r6_1d1a | MATα ura3-52 YBR209W::pGS62::ybr209w pkp1 | This study |
| **GSY2751** | C1-11r6_1d1b | MATa ura3-52 YBR209W::pGS62::ybr209w pkp1 | This study |
| **GSY2752** | C1-11r6_1d1c | MATα ura3-52 YBR209W::pGS62::ybr209w mth1-2 pkp1 | This study |
| **GSY2753** | C1-11r6_1d1d | MATa ura3-52 YBR209W::pGS62::ybr209w mth1-2 pkp1 | This study |
| **GSY2754** | C1-11r6_1d2a | MATα ura3-52 YBR209W::pGS62::ybr209w pkp1 | This study |
| **GSY2755** | C1-11r6_1d2b | MATa ura3-52 YBR209W::pGS62::ybr209w mth1-2 pkp1 | This study |
| **GSY2756** | C1-11r6_1d2c | MATα ura3-52 YBR209W::pGS62::ybr209w mth1-2 pkp1 | This study |
| **GSY2757** | C1-11r6_1d2d | MATa ura3-52 YBR209W::pGS62::ybr209w pkp1 | This study |
| **GSY2758** | C1-22y5_1a1a | MATa ura3-52 YBR209W::pGS64::ybr209w mot1 | This study |
| **GSY2759** | C1-22y5_1a1b | MATα ura3-52 YBR209W::pGS64::ybr209w mth1-3 mot1 | This study |
| **GSY2760** | C1-22y5_1a1c | MATα ura3-52 YBR209W::pGS64::ybr209w mot1 | This study |
| **GSY2761** | C1-22y5_1a1d | MATa ura3-52 YBR209W::pGS64::ybr209w mth1-3 mot1 | This study |
| **GSY2762** | C1-22y5_1a2a | MATa ura3-52 YBR209W::pGS64::ybr209w mot1 | This study |
| **GSY2763** | C1-22y5_1a2b | MATa ura3-52 YBR209W::pGS64::ybr209w mth1-3 mot1 | This study |
| **GSY2764** | C1-22y5_1a2c | MATα ura3-52 YBR209W::pGS64::ybr209w mot1 | This study |
| **GSY2765** | C1-22y5_1a2d | MATα ura3-52 YBR209W::pGS64::ybr209w mth1-3 mot1 | This study |
| **GSY2766** | C1-42y5_5b1a | MATa ura3-52 YBR209W::pGS64::ybr209w mot1 | This study |
| **GSY2767** | C1-42y5_5b1b | MATa ura3-52 YBR209W::pGS64::ybr209w muk1 mot1 | This study |
| **GSY2768** | C1-42y5_5b1c | MATα ura3-52 YBR209W::pGS64::ybr209w mot1 | This study |
| **GSY2769** | C1-42y5_5b1d | MATα ura3-52 YBR209W::pGS64::ybr209w muk1 mot1 | This study |
| **GSY2770** | C1-42y5_5b2a | MATa ura3-52 YBR209W::pGS64::ybr209w mot1 | This study |
| **GSY2771** | C1-42y5_5b2b | MATa ura3-52 YBR209W::pGS64::ybr209w muk1 mot1 | This study |
| **GSY2772** | C1-42y5_5b2c | MATα ura3-52 YBR209W::pGS64::ybr209w muk1 mot1 | This study |
| **GSY2773** | C1-42y5_5b2d | MATα ura3-52 YBR209W::pGS64::ybr209w mot1 | This study |
| **GSY2774** | C1-29r4_5a1a1a | MATα ura3-52 YBR209W::pGS62::ybr209w rim15 pkp1 | This study |
| **GSY2775** | C1-29r4_5a1a1b | MATa ura3-52 YBR209W::pGS62::ybr209w rim15 pkp1 | This study |
| **GSY2776** | C1-29r4_5a1a1c | MATa ura3-52 YBR209W::pGS62::ybr209w rim15 pkp1 | This study |
| **GSY2777** | C1-29r4_5a1a1d | MATa ura3-52 YBR209W::pGS62::ybr209w pkp1 | This study |
| **GSY2778** | C1-29r4_5a1a2a | MATa ura3-52 YBR209W::pGS62::ybr209w rim15 pkp1 | This study |
| **GSY2779** | C1-29r4_5a1a2b | MATα ura3-52 YBR209W::pGS62::ybr209w pkp1 | This study |
| **GSY2780** | C1-29r4_5a1a2c | MATa ura3-52 YBR209W::pGS62::ybr209w rim15 pkp1 | This study |
| **GSY2781** | C1-29r4_5a1a2d | MATα ura3-52 YBR209W::pGS62::ybr209w pkp1 | This study |
| **GSY2782** | C1-42y5_11c1d1a | MATa ura3-52 YBR209W::pGS64::ybr209w mot1 | This study |
| **GSY2783** | C1-42y5_11c1d1b | MATa ura3-52 YBR209W::pGS64::ybr209w sly41 mot1 | This study |
| **GSY2784** | C1-42y5_11c1d1c | MATα ura3-52 YBR209W::pGS64::ybr209w mot1 | This study |
| **GSY2785** | C1-42y5_11c1d1d | MATα ura3-52 YBR209W::pGS64::ybr209w sly41 mot1 | This study |
| **GSY2786** | C1-42y5_11c1d2a | MATa ura3-52 YBR209W::pGS64::ybr209w mot1 | This study |
| **GSY2787** | C1-42y5_11c1d2b | MATα ura3-52 YBR209W::pGS64::ybr209w mot1 | This study |
| **GSY2788** | C1-42y5_11c1d2c | MATa ura3-52 YBR209W::pGS64::ybr209w sly41 mot1 | This study |
| **GSY2789** | C1-42y5_11c1d2d | MATα ura3-52 YBR209W::pGS64::ybr209w sly41 mot1 | This study |
| **GSY2790** | C1-29r4_8d4b1a | MATa ura3-52 YBR209W::pGS62::ybr209w pkp1 | This study |
| **GSY2791** | C1-29r4_8d4b1b | MATα ura3-52 YBR209W::pGS62::ybr209w taf5 pkp1 | This study |
| **GSY2792** | C1-29r4_8d4b1c | MATa ura3-52 YBR209W::pGS62::ybr209w pkp1 | This study |
| **GSY2793** | C1-29r4_8d4b1d | MATα ura3-52 YBR209W::pGS62::ybr209w taf5 pkp1 | This study |
| **GSY2794** | C1-29r4_8d4b2a | MATα ura3-52 YBR209W::pGS62::ybr209w taf5 pkp1 | This study |
| **GSY2795** | C1-29r4_8d4b2b | MATα ura3-52 YBR209W::pGS62::ybr209w pkp1 | This study |
| **GSY2796** | C1-42y5_1b2b1b1a | MATα ura3-52 YBR209W::pGS64::ybr209w mot1 | This study |
| **GSY2797** | C1-42y5_1b2b1b1b | MATa ura3-52 YBR209W::pGS64::ybr209w vma8 mot1 | This study |
| **GSY2798** | C1-42y5_1b2b1b1c | MATα ura3-52 YBR209W::pGS64::ybr209w vma8 mot1 | This study |
| **GSY2799** | C1-42y5_1b2b1b1d | MATa ura3-52 YBR209W::pGS64::ybr209w mot1 | This study |
| **GSY2800** | C1-42y5_1b2b1b2a | MATα ura3-52 YBR209W::pGS64::ybr209w mot1 | This study |
| **GSY2801** | C1-42y5_1b2b1b2b | MATα ura3-52 YBR209W::pGS64::ybr209w vma8 mot1 | This study |
| **GSY2802** | C1-42y5_1b2b1b2c | MATa ura3-52 YBR209W::pGS64::ybr209w mot1 | This study |
| **GSY2803** | C1-42y5_1b2b1b2d | MATa ura3-52 YBR209W::pGS64::ybr209w vma8 mot1 | This study |
| **GSY2804** | C1-(11r6_1d1cx29r4_11c1a)_7b | MATa ura3-52 YBR209W::pGS62::ybr209w mth1-2 (HXT6/7)_red_ pkp1 | This study |
| **GSY2805** | C1-(11r6_1d1cx29r4_11c1a)_11b | MATa ura3-52 YBR209W::pGS62::ybr209w mth1-2 (HXT6/7)_red_ pkp1 | This study |
| **GSY2806** | C1-(11r6_1d1cx29r4_11c1a)_11a | MATα ura3-52 YBR209W::pGS62::ybr209w mth1-2 (HXT6/7)_red_ pkp1 | This study |
| **GSY2807** | C1-(11r6_1d1cx29r4_11c1a)_3c | MATa ura3-52 YBR209W::pGS62::ybr209w pkp1 | This study |
| **GSY2808** | C1-(11r6_1d1cx29r4_11c1a)_3d | MATα ura3-52 YBR209W::pGS62::ybr209w mth1-2 (HXT6/7)_red_ pkp1 | This study |
| **GSY2809** | C1-(11r6_1d1cx29r4_11c1a)_2c | MATα ura3-52 YBR209W::pGS62::ybr209w (HXT6/7)_red_ pkp1 | This study |
| **GSY2810** | C1-(11r6_1d1cx29r4_11c1a)_9c | MATa ura3-52 YBR209W::pGS62::ybr209w (HXT6/7)_red_ pkp1 | This study |
| **GSY2811** | C1-(11r6_1d1cx29r4_11c1a)_3c | MATa ura3-52 YBR209W::pGS62::ybr209w pkp1 | This study |
| **GSY2812** | C1-(11r6_1d1cx29r4_11c1a)_2b | MATα ura3-52 YBR209W::pGS62::ybr209w mth1-2 pkp1 | This study |
| **GSY2813** | C1-(11r6_1d1cx29r4_11c1a)_2d | MATa ura3-52 YBR209W::pGS62::ybr209w mth1-2 pkp1 | This study |
| **GSY2814** | C1-(11r6_1d1cx29r4_11c1a)_9d | MATα ura3-52 YBR209W::pGS62::ybr209w mth1-2 pkp1 | This study |
| **GSY2815** | C1-(22y5_1a1bx42y5_2c1c1b)_1a | MATα ura3-52 YBR209W::pGS64::ybr209w mth1-3 (HXT6/7)_yellow_ mot1 | This study |
| **GSY2816** | C1-(22y5_1a1bx42y5_2c1c1b)_2b | MATa ura3-52 YBR209W::pGS64::ybr209w mth1-3 (HXT6/7)_yellow_ mot1 | This study |
| **GSY2817** | C1-(22y5_1a1bx42y5_2c1c1b)_1b | MATa ura3-52 YBR209W::pGS64::ybr209w mot1 | This study |
| **GSY2818** | C1-(22y5_1a1bx42y5_2c1c1b)_3d | MATα ura3-52 YBR209W::pGS64::ybr209w mot1 | This study |
| **GSY2819** | C1-(22y5_1a1bx42y5_2c1c1b)_1d | MATa ura3-52 YBR209W::pGS64::ybr209w (HXT6/7)_yellow_ mot1 | This study |
| **GSY2820** | C1-(22y5_1a1bx42y5_2c1c1b)_2d | MATα ura3-52 YBR209W::pGS64::ybr209w (HXT6/7)_yellow_ mot1 | This study |
| **GSY2821** | C1-(22y5_1a1bx42y5_2c1c1b)_1c | MATα ura3-52 YBR209W::pGS64::ybr209w mth1-3 mot1 | This study |
| **GSY2822** | C1-(22y5_1a1bx42y5_2c1c1b)_2a | MATa ura3-52 YBR209W::pGS64::ybr209w mth1-3 mot1 | This study |
| **GSY2823** | D105 | MATα ura3-52 YBR209W::pGS62::ybr209w mth1-3 | This study |
| **GSY2824** | D106 | MATa ura3-52 YBR209W::pGS64::ybr209w mth1-2 | This study |
| **GSY2825** | D107 | MATa ura3-52 YBR209W::pGS62::ybr209w mth1-3 | This study |
| **GSY2826** | C1-(22y5_1b1dx42y5_1b2c1d)_2c | MATα ura3-52 YBR209W::pGS64::ybr209w ira1 gpb2 mot1 | This study |
| **GSY2827** | C1-(22y5_1b1dx42y5_1b2c1d)_1a | MATa ura3-52 YBR209W::pGS64::ybr209w mot1 | This study |
| **GSY2828** | C1-(22y5_1b1dx42y5_1b2c1d)_3c | MATα ura3-52 YBR209W::pGS64::ybr209w ira1 mot1 | This study |
| **GSY2829** | C1-(22y5_1b1dx42y5_1b2c1d)_6c | MATa ura3-52 YBR209W::pGS64::ybr209w gpb2 mot1 | This study |
| **GSY2830** | C1-(29r4_5a1a2ax42y5_1b2c1b)_1d | MATa ura3-52 YBR209W::pGS64::ybr209w rim15 | This study |
| **GSY2831** | C1-(29r4_5a1a2ax42y5_1b2c1b)_2c | MATa ura3-52 YBR209W::pGS64::ybr209w rim15 gpb2 | This study |
| **GSY2832** | C1-(29r4_5a1a2ax42y5_1b2c1b)_10c | MATa ura3-52 YBR209W::pGS64::ybr209w gpb2 | This study |
| **GSY2833** | C1-(29r4_5a1a2ax42y5_1b2c1b)_1a | MATa ura3-52 YBR209W::pGS64::ybr209w pkp1 | This study |
| **GSY2834** | C1-(22y5_1b1dx42y5_2c1c1b)_2c | MATα ura3-52 YBR209W::pGS64::ybr209w mot1 | This study |
| **GSY2835** | C1-(22y5_1b1dx42y5_2c1c1b)_1c | MATa ura3-52 YBR209W::pGS64::ybr209w ira1 (HXT6/7)_yellow_ mot1 | This study |
| **GSY2836** | C1-(22y5_1b1dx42y5_2c1c1b)_1a | MATa ura3-52 YBR209W::pGS64::ybr209w (HXT6/7)_yellow_ mot1 | This study |
| **GSY2837** | C1-(22y5_1b1dx42y5_2c1c1b)_1b | MATα ura3-52 YBR209W::pGS64::ybr209w ira1 mot1 | This study |
| **GSY2838** | C1-(22y5_1b1dx29r4_5a1a2a)_2a | MATa ura3-52 YBR209W::pGS64::ybr209w ira1 rim15 | This study |
| **GSY2839** | C1-(22y5_1b1dx29r4_5a1a2a)_7d | MATα ura3-52 YBR209W::pGS64::ybr209w | This study |
| **GSY2840** | C1-(22y5_1b1dx29r4_5a1a2a)_10c | MATα ura3-52 YBR209W::pGS64::ybr209w rim15 | This study |
| **GSY2841** | C1-(22y5_1b1dx29r4_5a1a2a)_1b | MATa ura3-52 YBR209W::pGS62::ybr209w ira1 | This study |
| **GSY2842** | C1-(22y5_1a1bx42y5_1b2c1d)_2c | MATα ura3-52 YBR209W::pGS64::ybr209w mth1-3 gpb2 mot1 | This study |
| **GSY2843** | C1-(22y5_1a1bx42y5_1b2c1d)_6a | MATa ura3-52 YBR209W::pGS64::ybr209w mth1-3 mot | This study |
| **GSY2844** | C1-(22y5_1a1bx42y5_1b2c1d)_6b | MATα ura3-52 YBR209W::pGS64::ybr209w gpb2 mot1 | This study |
