# Supplementary material for: Reciprocal Sign Epistasis between Frequently Experimentally Evolved Adaptive Mutations Causes a Rugged Fitness Landscape
Source: PLoS Genet. 2011 Apr 28;7(4):e1002056. doi: 10.1371/journal.pgen.1002056 (PMC3084205; doi:10.1371/journal.pgen.1002056)
Supplement: Table S4 — Primers used. Usage key: A – allele-specific PCR, B – mutation confirmation by Sanger sequencing, C – quantitative Sanger sequencing, D – quantitative PCR. (DOC) [file pgen.1002056.s010.doc]

| **Use** | **Primer name** | **Targeted feature** | **Sequence** |
| --- | --- | --- | --- |
| A | asPCR_C1-HXT6for | *HXT6/7* | **CAATGGCTTATCATCGTGACC** |
| A | asPCR_C1_HXT7p_rev | *HXT6/7* | **GGAAAAATCCCCACCATCTT** |
| A | asPCR_C1-green_ch4_com_rev | *mth1-1* | **GGATCTGTACATGGAGGCTA** |
| A | asPCR_C1-green_ch4_mut_for | *mth1-1* | **TTTTAAGTTGGACTGTGA** |
| A | asPCR_C1-green_ch4_wt_for | *mth1-1* | **TTTTAAGTTGGACTGTGG** |
| A | asPCR_C1-red_ch11_com_for | *mnn4* | **TTTCTGTTAACGTCGGATAAA** |
| A | asPCR_C1-red_ch11_mut_rev | *mnn4* | **CAGACTACATTAGATCAAGTTACAG** |
| A | asPCR_C1-red_ch11_wt_rev | *mnn4* | **CAGACTACATTAGATCAAGTTACAA** |
| A | asPCR_C1-red_ch15LTR_com_rev | chr15:301008 | **GCTACGTCTGACCGAATACT** |
| A | asPCR_C1-red_ch15LTR_mut_for | chr15:301008 | **CAATAATAAGTGGATGGTACTGT** |
| A | asPCR_C1-red_ch15LTR_wt_for | chr15:301008 | **CAATAATAAGTGGATGGTACTGG** |
| A | asPCR_C1-red_ch16_com_for | chr16:912523 | **TTGGAAAGCATCTTTGAGTT** |
| A | asPCR_C1-red_ch16_mut_rev | chr16:912523 | **AAACTATACAATGCACAGCCC** |
| A | asPCR_C1-red_ch16_wt_rev | chr16:912523 | **AAACTATACAATGCACAGCCA** |
| A | asPCR_C1-red_ch2_com_for | *taf5* | **CCGTCATTTCTATTGCTGTT** |
| A | asPCR_C1-red_ch2_mut_rev | *taf5* | **AATAAATGGCATTCTTGA** |
| A | asPCR_C1-red_ch2_wt_rev | *taf5* | **AATAAATGGCATTCTTGC** |
| A | asPCR_C1-red_ch4MTH1_com_for | *mth1-2* | **TATTCGGAGCATCTTTGTTT** |
| A | asPCR_C1-red_ch4MTH1_mut_rev | *mth1-2* | **ATGGAGGCTAACTTAGATTACT** |
| A | asPCR_C1-red_ch4MTH1_wt_rev | *mth1-2* | **ATGGAGGCTAACTTAGATTACG** |
| A | asPCR_C1-red_ch7_com_for | *cox18* | **TGTAGTTGTAACGGCATGAA** |
| A | asPCR_C1-red_ch7_mut_rev | *cox18* | **TATACCATGGATAGTGGA** |
| A | asPCR_C1-red_ch7_wt_rev | *cox18* | **TATACCATGGATAGTGGT** |
| A | asPCR_C1-red_PKP1_comFor | *pkp1* | **TACGTCTTGCACATTGGTAG** |
| A | asPCR_C1-red_PKP1_mutRef | *pkp1* | **GAGCTGTACTTACGGATACGAT** |
| A | asPCR_C1-red_PKP1_wtRev | *pkp1* | **GAGCTGTACTTACGGATACGAG** |
| A | asPCR_C1-yellow_ch11BYE1_com_rev | *bye1* | **TGTCTGTCCGTACTTCTTCA** |
| A | asPCR_C1-yellow_ch11BYE1_mut_for | *bye1* | **TCGTCTGTTCTTACATTTTGG** |
| A | asPCR_C1-yellow_ch11BYE1_wt_for | *bye1* | **TCGTCTGTTCTTACATTTTGA** |
| A | asPCR_C1-yellow_ch15_com_for | *sly41* | **GACGTCATCCGTAGAAGAAG** |
| A | asPCR_C1-yellow_ch15_mut_rev | *sly41* | **GCGTTATGACTACTTGCCT** |
| A | asPCR_C1-yellow_ch15_wt_rev | *sly41* | **GCGTTATGACTACTTGCCG** |
| A | asPCR_C1-yellow_ch2_com_rev | *ira1* | **TGTCAAGACCATTAAGGGAA** |
| A | asPCR_C1-yellow_ch2_mut_for | *ira1* | **CCAAATGATACTGTAGAAGAGT** |
| A | asPCR_C1-yellow_ch2_wt_for | *ira1* | **CCAAATGATACTGTAGAAGAGC** |
| A | asPCR_C1-yellow_ch4MTH1_com_for | *mth1-3* | **CGATGATATTTCTCCATTCC** |
| A | asPCR_C1-yellow_ch4MTH1_mut_rev | *mth1-3* | **GCCCCAGTTTAGGTTACAACA** |
| A | asPCR_C1-yellow_ch4MTH1_wt_rev | *mth1-3* | **GCCCCAGTTTAGGTTACAACT** |
| A | asPCR_C1-yellow_ch5VMA8_com_for | *vma8* | **AAGGTTAGATGAAGCAGCAG** |
| A | asPCR_C1-yellow_ch5VMA8_mut_rev | *vma8* | **CTGAATCTCTTTGTTAGGGCGG** |
| A | asPCR_C1-yellow_ch5VMA8_wt_rev | *vma8* | **CTGAATCTCTTTGTTAGGGCGT** |
| A | asPCR_C1-yellow_ch9DAL81_com_rev | *dal81* | **CAGTTCCTTTGGAGTTTGAG** |
| A | asPCR_C1-yellow_ch9DAL81_mut_for | *dal81* | **TTGAACAGGTATTGAAACTCA** |
| A | asPCR_C1-yellow_ch9DAL81_wt_for | *dal81* | **TTGAACAGGTATTGAAACTCG** |
| A | Chr1_yellow_for (GPB2 asPCR) | *gpb2* | **GGGCATGCGTTGAAGAATCC** |
| A | Chr1_yellow_rev (GPB2 asPCR) | *gpb2* | **GACCGCACAGTACGATTCAG** |
| A | Chr16_yellow_mut_for (MUK1 asPCR) | *muk1* | **CAGTAGAGTCTCATTGCCATA** |
| A | Chr16_yellow_rev (MUK1 asPCR) | *muk1* | **CTTTGGATAGTAGGAGTCACG** |
| A | Chr16_yellow_wt_for (MUK1 asPCR) | *muk1* | **CAGTAGAGTCTCATTGCCATC** |
| A | Chr6_red_mut_for (RIM15 asPCR) | *rim15* | **TGAAATGGTTCCTGATCTTTACA** |
| A | Chr6_red_rev (RIM15 asPCR) | *rim15* | **GGAATTGATCTAACAAAGCATTG** |
| A | Chr6_red_wt_for (RIM15 asPCR) | *rim15* | **GAAATGGTTCCTGATCTTTACC** |
| B | seqPCR_C1-allRed_ch9_for | *pkp1* | **CTTGCCCCCAAACAGTTCTA** |
| B | seqPCR_C1-allRed_ch9_rev | *pkp1* | **CCAGACGATGACGAGCTGTA** |
| B | seqPCR_C1-green_ch2_Int_353579_2_for | chr02:353579 | **TGCAAAATAGCTAATGGAAATGG** |
| B | seqPCR_C1-green_ch2_Int_353579_2_rev | chr02:353579 | **GATGCTAAAAGAACGTTTACACACA** |
| B | seqPCR_C1-red_ch15LTRfor | chr15:301008 | **TTCCACCACGAACCGTAGAT** |
| B | seqPCR_C1-red_ch15LTRrev | chr15:301008 | **TGCGTACACATGCTACATCG** |
| B,C | seqPCR_C1-red_ch4MTH1_for | *mth1-2; mth1-3* | **TGCATTGTGCCTCTACTGCT** |
| B,C | seqPCR_C1-red_ch4MTH1_rev | *mth1-2; mth1-3* | **GCAGACCCATCCAACATTCT** |
| B | seqPCR_C1-yel_ch11_BYE1_for | *bye1* | **CCATCGCACTGTACCATGTC** |
| B | seqPCR_C1-yel_ch11_BYE1_rev | *bye1* | **AGGAGGAGACTGAAGCACCA** |
| B | seqPCR_C1-yel_ch16_MOT1_for | *mot1* | **GCTTCTGTTTCGCCTTGAAC** |
| B | seqPCR_C1-yel_ch16_MOT1_rev | *mot1* | **GCATCCAGAAGGGGAAAAAT** |
| B | seqPCR_C1-yel_ch5_VMA8_for | *vma8* | **TATGACGCTGGGTTTGATGA** |
| B | seqPCR_C1-yel_ch5_VMA8_rev | *vma8* | **GAAAAGGCAGCAGTTTGCAT** |
| B | seqPCR_C1-yel_ch9_DAL81_for | *dal81* | **CTTGGGGAAATCATGGACAC** |
| B | seqPCR_C1-yel_ch9_DAL81_rev | *dal81* | **ACGTAATTTCCGTGGCAAAA** |
| B | Chr1_39603_for | *gpb2* | **GATTGTGATTCATTGGCAGG** |
| B | Chr1_39603_rev | *gpb2* | **TCCTTCCTTAGCTTTGGTAC** |
| B | Chr11_64696_for | *mnn4* | **AGGGTCCTTATCAAACATTTG** |
| B | Chr11_64696_rev | *mnn4* | **TTTAGATTTTGGTTCGACTATG** |
| B | Chr15_893328_for | *sly41* | **AGGGCTACTGTTTCTAGCG** |
| B | Chr15_893328_rev | *sly41* | **TGTTGGATTTGCTTCCATCG** |
| B | Chr16_422264_for | *muk1* | **TGAAACTACATGAAATCATGAC** |
| B | Chr16_422264_rev | *muk1* | **AGATGCTGAAGATCGTGAAG** |
| B | Chr16_912519_for | chr16:912523 | **TTCTTCCCCATGATAGATAAG** |
| B | Chr16_912519_rev | chr16:912523 | **CATCATACGAAAGTATGTCTG** |
| B | Chr2_521872_for | *ira1* | **AAACGTTGACATTGGCATTAG** |
| B | Chr2_521872_rev | *ira1* | **AGCACTCTGAAAAGAACCTG** |
| B | Chr2_616426_for | *taf5* | **GAACGACGCTACTAAATCGC** |
| B | Chr2_616426_rev | *taf5* | **GTTTCTCCCCGCTAGGCC** |
| B | Chr4_1014688_for | *mth1-1* | **CCTTGGGAATTTGGAGCTC** |
| B | Chr4_1014688_rev | *mth1-1* | **GAATTCAAACTAACCAGCGC** |
| B | Chr6_73424_for | *rim15* | **CTAGTCCAGGATCCTGATC** |
| B | Chr6_73424_rev | *rim15* | **CTTGGAAGCACGTGGTATC** |
| B | Chr7_617106_for | *cox18* | **TTCTGCCTCTTTCTTGTCTC** |
| B | Chr7_617106_rev | *cox18* | **TGAGGTTGTGGAGCTGTTG** |
| D | qPCR_control_chr04_UBP1_for | *UBP1* | **GCGCTCTGTCATTGTTCACT** |
| D | qPCR_control_chr04_UBP1_rev | *UBP1* | **GACTTCAGCTTCGTCCACAA** |
| D | qPCR_HXT6.7coding_chr04_1159850_1161350_for | *HXT6/7* | **TTCGTGCTCTTCACCTTCAC** |
| D | qPCR_HXT6.7coding_chr04_1159850_1161350_rev | *HXT6/7* | **TCCTGTGGAGCATCTCTCTG** |
| D | qPCR_HXT6.7promoter_chr04_1157000_1159000 | *HXT6/7* | **GATTATTGCTGGTCCGATCC** |
| D | qPCR_HXT6.7promoter_chr04_1157000_1159000 | *HXT6/7* | **GAGTAATCGCCAATGGGTCT** |
